# Human monoclonal antibodies against chikungunya virus target multiple distinct epitopes in the E1 and E2 glycoproteins

**DOI:** 10.1371/journal.ppat.1008061

**Published:** 2019-11-07

**Authors:** Jose A. Quiroz, Ryan J. Malonis, Larissa B. Thackray, Courtney A. Cohen, Jesper Pallesen, Rohit K. Jangra, Rebecca S. Brown, Daniel Hofmann, Frederick W. Holtsberg, Sergey Shulenin, Elisabeth K. Nyakatura, Lorellin A. Durnell, Vinayak Rayannavar, Johanna P. Daily, Andrew B. Ward, M. Javad Aman, John M. Dye, Kartik Chandran, Michael S. Diamond, Margaret Kielian, Jonathan R. Lai

**Affiliations:** 1 Department of Biochemistry, Albert Einstein College of Medicine, Bronx, New York, United States of America; 2 Department of Medicine, Washington University in St. Louis, School of Medicine, St. Louis, Missouri, United States of America; 3 Virology Division, United States Army Medical Research Institute of Infectious Diseases, Fort Detrick, Maryland, United States of America; 4 Department of Integrative Structural and Computational Biology, The Scripps Research Institute, La Jolla, California, United States of America; 5 Department of Microbiology and Immunology, Albert Einstein College of Medicine, Bronx, New York, United States of America; 6 Department of Cell Biology, Albert Einstein College of Medicine, Bronx, New York, United States of America; 7 Integrated Biotherapeutics Inc., Rockville, Maryland, United States of America; 8 Department of Medicine, Albert Einstein College of Medicine, Bronx, New York, United States of America; 9 Department of Molecular Microbiology, Washington University in St. Louis, School of Medicine, St. Louis, Missouri, United States of America; 10 Department of Pathology & Immunology, Washington University in St. Louis, School of Medicine, St. Louis, Missouri, United States of America; Icahn School of Medicine at Mount Sinai, UNITED STATES

## Abstract

Chikungunya virus (CHIKV) is a mosquito-transmitted alphavirus that causes persistent arthritis in a subset of human patients. We report the isolation and functional characterization of monoclonal antibodies (mAbs) from two patients infected with CHIKV in the Dominican Republic. Single B cell sorting yielded a panel of 46 human mAbs of diverse germline lineages that targeted epitopes within the E1 or E2 glycoproteins. MAbs that recognized either E1 or E2 proteins exhibited neutralizing activity. Viral escape mutations localized the binding epitopes for two E1 mAbs to sites within domain I or the linker between domains I and III; and for two E2 mAbs between the β-connector region and the B-domain. Two of the E2-specific mAbs conferred protection *in vivo* in a stringent lethal challenge mouse model of CHIKV infection, whereas the E1 mAbs did not. These results provide insight into human antibody response to CHIKV and identify candidate mAbs for therapeutic intervention.

## Author summary

Chikungunya virus (CHIKV) is a globally emerging virus that can cause significant disease, including a prolonged and painful arthritis. The virus is spread by mosquitoes that circulate in many regions of the world including the United States. Currently, there are no available vaccines or therapies to treat CHIKV infection. In this report, we identified and characterized a large panel of antibodies against CHIKV from two donors that contracted the viral infection in the Dominican Republic. These antibodies target a number of different regions of the membrane proteins that coat the surface of the virus, and many can inhibit the ability of CHIKV to infect cells. Two of the antibodies were shown to protect mice from a lethal dose of CHIKV. These antibodies have therapeutic potential, and provide insight into the human immune response that may facilitate vaccine development.

## Introduction

Chikungunya virus (CHIKV) is a member of the alphavirus genus in the *Togaviridae* family of positive-stranded RNA viruses. Alphaviruses are transmitted by mosquito vectors and cause severe human and animal illness [1]. CHIKV usually infects human musculoskeletal tissues and results in a painful polyarthritis that can persist for years. In rare cases (~0.1%), CHIKV can cause mortality, especially in neonates and the elderly [2, 3]. CHIKV was discovered in Africa where it is endemic and causes large but sporadic outbreaks. Beginning in 2004, CHIKV emerged to cause a multi-year pandemic in countries around the Indian Ocean, with millions of reported cases [3]. The first contemporary report of CHIKV in the Americas occurred in 2013, followed by rapid spread of the virus to cause an estimated 1.8 million cases in over 43 countries [4, 5]. There are three genotypes of CHIKV (Asian, East/Central/South African (ECSA), and West African) that are ~92.5–98% identical at the amino acid level. Global spread of some CHIKV strains was precipitated by adaptation of the envelope glycoprotein to allow efficient transmission from both *Aedes aegypti* and *Aedes albopictus* mosquitoes [6]. These mosquitos also transmit globally important pathogens including the flaviviruses, Dengue virus (serotypes 1–4, DENV-1 to -4), yellow fever virus (YFV), and Zika virus (ZIKV) [7]. Both *Aedes* species of mosquitos are found in the continental U.S., with A. *aegypti* circulating in the Panhandle states (Florida, Mississippi, Alabama, Louisiana, and Texas) and *A*. *albopictus* reaching as far north as New York.

**Fig 1:**
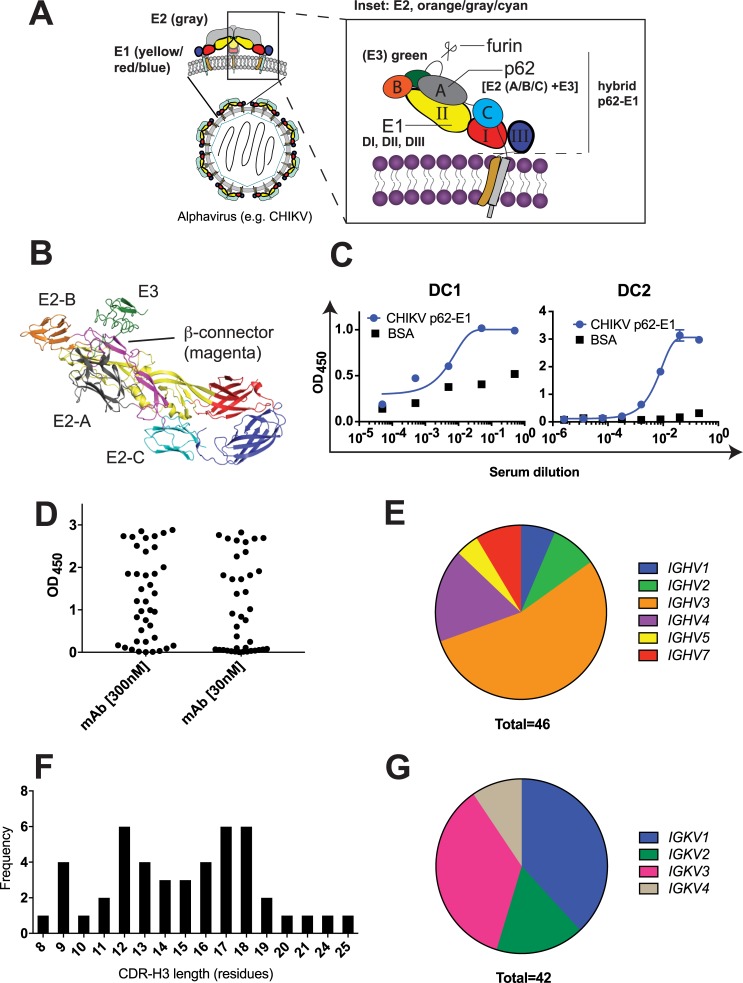
Chikungunya virus glycoprotein architecture and overview of human monoclonal antibodies. (**A**) The CHIKV glycoprotein spike consists of three copies each of E1 and E2 each in the prefusion form (modified from [8]). The inset shows the arrangement of E1 (comprised of DI, DII, and DIII) and E2 (comprised of domains A, B, and C). The location of E3 and the furin cleavage site also are shown. The hybrid protein “p62-E1”, consisting of p62 ectodomain linked to the E1 ectodomain by a polypeptide linker, was used for binding and sorting experiments. (**B**) X-ray crystallographic structure of CHIKV p62-E1 (PDB ID: 3N40) [9] with domains colored according to panel A, and with the β-connector colored magenta. (**C**) Reactivity of plasma from convalescent patients DC1 and DC2 toward CHIKV p62-E1 in comparison to negative control wells (3% BSA). A representative dataset is shown for the DC2 ELISA from two experiments, each performed in triplicate (points represent mean ± SD). Sera for DC1 were limited and thus data presented here are from a single experiment with no replicates. (**D**) Volcano plot of ELISA (OD_450_) for 46 of the isolated mAbs at 30 nM and 300 nM. Each data point represents the mean from 2 or more replicates. Distribution of IGHV families (**E**), CDR-H3 lengths (**F**), and IGKV families (**G**) for the mAbs.

The two alphavirus envelope glycoproteins E1 and E2, each containing a single transmembrane domain, are responsible for mediating viral attachment (E2) and membrane fusion (E1) (**Fig 1A**) [1, 8, 9]. The prefusion E1/E2 heterodimer is arranged in 80 trimeric spikes with an icosahedral organization on the CHIKV particle [10, 11]. Mature E1/E2 is generated by furin cleavage of a penultimate precursor that consists of a non-covalent heterodimer of E1 and p62. The p62 polypeptide contains E2 and E3, the latter of which is a small domain that accompanies the glycoprotein throughout virus biogenesis and prevents premature conformational changes and fusion [12, 13]. Furin cleavage in the region between E2 and E3 releases E3 and primes the glycoprotein for low pH-triggered membrane fusion during virus entry [1, 8, 9, 13, 14].

The X-ray crystallography structure of a hybrid “p62-E1” protein consisting of the soluble ectodomains of p62 and E1 joined by a polypeptide linker, revealed that the E1/E2 heterodimer forms a “twisted plate” architecture (**Fig 1B**) [9]. E2 contains three globular domains, A, B, and C, as well as a β-ribbon connector region (also known as the “β-connector” or “β-arch”) that runs lengthwise across the E2 ectodomain and links the membrane-distal B domain with the central A domain and membrane-proximal C domain. The E1 subunit contains primarily β-sheet structure that is reminiscent of the flavivirus E protein and other structurally-defined “class II” viral glycoproteins, with three domains, DI, DII, and DIII. DII contains the fusion loop that inserts into the host cell membrane during fusion, and DI acts as a central hinge that leads into DIII, which is the most mobile region during viral membrane fusion. In the prefusion structure of the mature viral particle, the E2 subunit constitutes the majority of the accessible viral surface, consistent with its role in cell attachment. The recently published cryo-EM structures of CHIKV in complex with the ectodomains of Mxra8, a newly-identified alphavirus receptor[15], revealed that Mxra8 engages the trimeric E1/E2 spike with “low” and “high” affinity binding sites [16, 17]. The high affinity site is thought to be most relevant to viral entry; in this binding mode, the D1 and D2 domains of Mxra8 are inserted into a cleft between E1 and E2 with major contacts to the A and B domains of E2 and the fusion loop of E1. A number of neutralizing E2 antibodies target epitopes in these regions.

Currently, there is no licensed vaccine or therapy for CHIKV infection, although several candidates are currently under development [18–21]. An attenuated virus vaccine (181/25), derived from the AF15561 Asian strain by serial passage in human lung cells, ultimately did not advance due to reactogenicity and safety concerns [22]. Nonetheless, the CHIKV 181/25 strain is utilized in research settings because it can be handled under biosafety level 2 (BSL2) protocols. Monoclonal antibodies (mAbs) represent an attractive platform for development of prophylactic or therapeutic agents against viral diseases. Human or humanized mAbs are generally well-tolerated and have favorable pharmacokinetic profiles that can be extended up to several months with Fc engineering [23]. A number of murine and human mAbs targeting CHIKV have been described [24–31]. One combination of chimeric murine-human mAbs, CHK-152 (E2-specific) and CHK-166 (E1-specific) afforded high levels of protection in immunocompromised mice and reduced viremia in rhesus macaques [28]. Furthermore, the CHK-152/CHK-166 combination therapy reduced viral load in muscle and joint tissues of the extremities [29]. Recently, a recombinant mAb SINV001, which contains complementarity-determining regions (CDRs) of human-derived antibody 4N12 grafted onto a synthetic framework, was shown to reduce the viral load in joint tissues of non-human primates [24, 32].

Here, we describe the isolation of a panel of human mAbs from CHIKV-infected individuals in the convalescent phase by single B cell sorting. A number of these mAbs strongly neutralized infection *in vitro*, and targeted multiple epitopes in both the E1 and E2 subunits, as established by binding/competition, immunoprecipitation, and virus neutralization escape studies. Two E2-specific (DC2.429 and DC2.271B) and two E1-specific (DC1.7 and DC2.315) mAbs were evaluated in a stringent protection model in which 3-week old mice were administered a blocking anti-interferon (IFN) receptor mAb prior to lethal viral challenge with the La Reunion (ECSA) CHIKV strain LR2006_OPY1. The E2 mAbs conferred protection in this *in vivo* protection model, whereas the E1 mAbs did not. One E2 mAb (DC.271B) was highly protective after both prophylactic (80% survival) and therapeutic (100% survival) administration. Together, our results help to define the human immune response to CHIKV infection and provide human mAbs for potential therapeutic development.

## Results

### Single B-cell cloning and screening of CHIKV human mAbs

Plasma from two convalescent donors (DC1 and DC2) was tested for their capacity to bind recombinant CHIKV p62-E1 protein (**Fig 1A and 1B**). Both donors were exposed to CHIKV in the Dominican Republic (*D*ominican Republic, *C*hikungunya) within two years of sample collection and experienced fever, joint pain, and in the case of DC2, persistent arthritis. In both cases, serum reactivity against p62-E1 was observed relative to wells coated with BSA, indicating the presence of circulating CHIKV-specific antibodies (**Fig 1C**).

Peripheral blood mononuclear cells (PBMCs) from both patients were isolated and sorted for individual p62-E1-reactive B cells by fluorescence activated cell sorting (FACS). P62-E1 was chosen as the sorting antigen because it can be efficiently expressed in and purified from *Drosophila* S2 cells. Furthermore, previous isolation of human CHIKV antibodies via hybridoma methods resulted in the isolation of numerous mAbs that bind in the β-connector region of E2 [31], part of which lies underneath E3 in p62-E1. Thus, we reasoned that use of p62-E1 as a sorting antigen, in which parts of the β-connector were occluded by E3, might favor isolation of antibodies that target previously unrecognized epitopes of the glycoprotein. PBMCs were sorted for viability and size/granularity consistent with single lymphocytes. These populations were then negatively gated for T cells, macrophages, and other lymphocytes (CD3^+^/CD8^+^/CD14^+^); followed by positive gating for CD20^hi/lo^ CD27^+^ IgG^+^ p62-E1^+^ B cells (**S1 Fig**).

B cells that met these criteria were sorted into individual wells (generally less than 0.1% of PBMCs per sorting sample), lysed, and cDNA was generated and used for nested PCR with human-specific degenerate primers to recover variable domains of immunoglobulin heavy and light chains [33]. We focused exclusively on κ light chains due to their high abundance in natural human antibody repertoires and generally favorable biochemical properties [34]. The recovered variable domains were cloned and expressed as recombinant human IgG1 antibodies.

From 108 cloned human mAbs, we focused our analysis on 46 mAbs due to favorable functional and/or expression properties. All 46 of these mAbs were subjected to an ELISA against p62-E1 using 30 and 300 nM concentrations of mAb (**Fig 1D**). The mAbs bound p62-E1 with a range of OD_450_ values from 0 (non-binding) to 3 (high binding), but a number of mAbs exhibited strong reactivity (OD_450_ > 1.5) even at the lower concentration (30 nM). The binding was specific as none of the mAbs showed any significant binding to control wells coated with 3% BSA at 300 nM mAb concentration **(S2 Fig).** Sequence analysis revealed that the majority of the mAbs belonged to the IGHV3 family, consistent with the prevalence of this family in human repertoires; other IGHV families (1, 2, 4–7) comprised the remainder of the mAb population (**Fig 1E**). The CDR-H3 lengths (Kabat numbering) ranged from 8–25, with a bimodal distribution at 12 and 17–18 residues (**Fig 1F**). The light chains were distributed among IGKV1-4 families, with the IGKV1 and IGKV3 families each representing almost half (16/42 and 15/52, respectively; four light chain sequences were shared by pairs of heavy chains, thus bringing the total to 42 distinct VK sequences) (**Fig 1G**). The diversity of IGHV and IGKV families, as well as CDR-H3 lengths suggests a range of potential modes of interaction between mAbs and the CHIKV glycoprotein.

### Binding profiles and epitope binning

**Fig 2:**
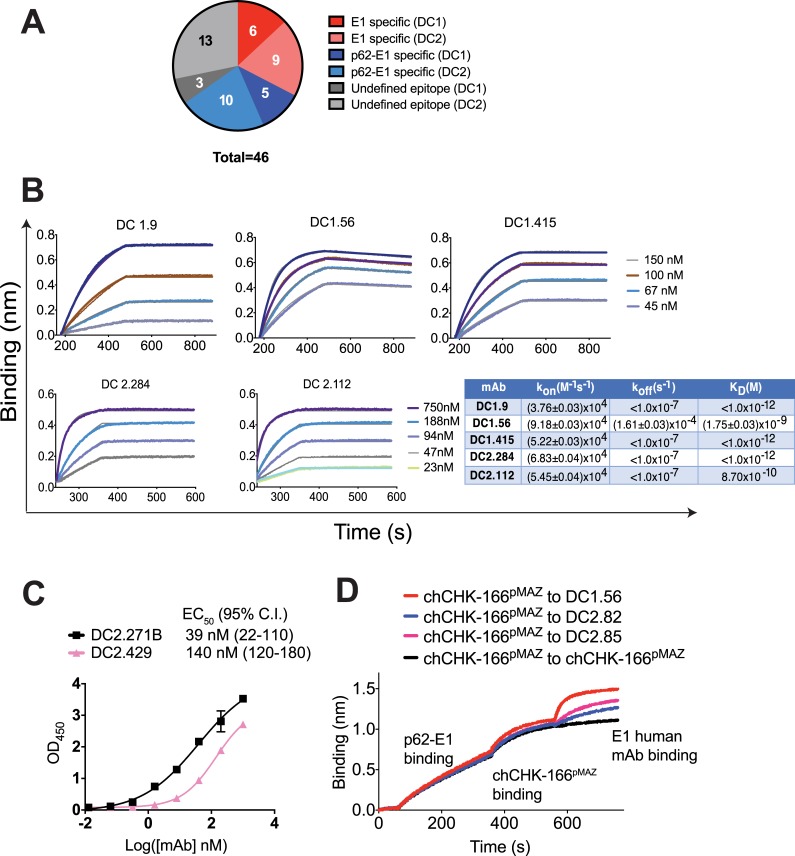
MAb binding profiles and epitope binning. (**A**) Distribution of specificities for mAbs isolated from DC1 and DC2, based on IP, ELISA and/or BLI studies. “p62-E1” specificity refers to mAbs that were confirmed to bind p62-E1 by ELISA and/or BLI, or that immunoprecipitated p62-E1 or E2. These mAbs likely have epitopes contained in E2 or shared epitopes across E1 and E2. “E1” specificity refers to mAbs that were confirmed to bind E1' by ELISA and/or BLI. (**B**) Binding of E1-specific mAbs to E1' by BLI. A representative dataset from two experiments is shown. (**C**) Full ELISA binding curves for DC2.271B and DC2.429 against p62-E1; a representative dataset from two experiments each performed in triplicate is shown (points represent mean ± SD). (**D**) Two-phase binding by BLI of E1 mAbs DC1.56, DC2.82, and DC2.85 against E1 mAb chCHK-166^pMAZ^. In all cases, the human mAbs were able to engage p62-E1 simultaneously as CHK-166, regardless of order of addition, thus indicating that they do not share epitopes with CHK-166. A representative dataset from two experiments is shown.

A combination of methods was used to bin the epitopes of the 46 mAbs (**Fig 2A, S3 Fig, S4 Fig and Table 1**). Immunoprecipitation (IP) experiments with lysates of radiolabeled virus-infected cells revealed a number of reactivity profiles, with some mAbs targeting E1 alone (11 mAbs) and others targeting a combination of p62 (containing both E2 and E3) and E2, with or without E1 (9 mAbs). For unknown reasons, a number of mAbs did not result in an IP signal. For many of the E1-specific mAbs, binding to the E1 subunit was confirmed by ELISA against an S2-expressed E1 ectodomain construct (E1'). Similarly, the majority of p62-E1-specific mAbs exhibited strong reactivity toward p62-E1 by ELISA, including several mAbs (*e*.*g*., DC1.43, DC2.12, DC2.432. and DC2.446) that lacked E1 reactivity and therefore likely engage epitopes completely contained on E2. Although murine mAbs targeting CHIKV E1 have been described [28], no human E1-specific CHIKV antibodies have been reported. E2 is thought to be the predominant antigenic target of antibodies that arise in response to infection. Cryo-electron microscopy (cryoEM) studies of CHIKV virus-like particles (VLPs) suggest that E1 is not abundantly exposed in the prefusion form [10, 11]. Nonetheless, we demonstrate here that E1 antibodies are elicited in response to natural human infection.

**Table 1:** Summary of immunoprecipitation (IP) and E1’ or p62-E1 ELISA results^a^.

E1-specific				p62-E1 specific		
mAb	IP	E1’ ELISA	p62-E1 ELISA	mAb	IP	p62-E1 ELISA
DC1.7	E1	+	+	DC1.33	No IP	+
DC1.9	E1	ND	+	DC1.43	E2 + p62	+
DC1.55	No IP	+	+	DC1.159	E1, E2, p62	ND
DC1.56	E1	+	+	DC1.364	E1, E2, p62	ND
DC1.355	E1	ND	ND	DC2.1	E2 + p62	+
DC1.380	E1	ND	ND	DC2.3	E1, E2, p62	+
DC2.23	E1^b^	ND	+	DC2.12	E2 + p62	+
DC2.74	No IP	+	+	DC2.80	ND	+
DC2.82	E1	+	+	DC2.95	ND	+
DC2.112	E1	+	+	DC2.118	No IP	+
DC2.131	E1	ND	+	DC2.134	No IP	+
DC2.284	E1	+	+	DC2.148	No IP	+
DC2.315	E1	+	+	DC2.159	E1, E2, p62	+
				DC2.271B	No IP	+
				DC2.422	ND	+
				DC2.429	No IP	+
				DC2.432	E2 + p62	+
				DC2.446	E2 + p62	ND
				DC2.502	No IP	+
				DC2.507	No IP	+
				DC2.541	No IP	+
				DC2.547	ND	+
				DC2.572	No IP	+
				DC2.580	No IP	+

^a^ All results confirmed by ≥ 2 independent experiments unless marked otherwise.

^b^ Experiment performed once.

+ Positive ELISA signal at 300 nM [mAb]

ND not determined

Detailed analysis was performed on 37 of the mAbs that had favorable expression yields and properties.

The binding affinity of a subset of mAbs was examined by biolayer interferometry (BLI) or full 8-point ELISA curves. The E1 mAbs DC1.9, DC1.56, DC1.415, DC2.284, and DC2.112 bound E1' with subnanomolar affinity, due to slow off-rates (k_off_ ~ 10^−7^–10^−4^ s^-1^) (**Fig 2B and S4 Fig**). An ELISA of p62-E1-specific mAbs DC2.271B and DC2.429 indicate that reactivity to p62-E1 is moderate, since the binding curves for DC2.271B and DC2.429 were relatively non-cooperative and had EC_50_ values in mid-nanomolar range (39 and 140 nM, respectively, **Fig 2C**), in comparison to E1-specific mAbs whose EC_50_ values were in single-digit nanomolar range or below (**S4 Fig**). As DC2.271B and DC2.429 were among the most potent neutralizing mAbs (see below); their binding to the soluble p62-E1 does not directly correlate with neutralizing potency and more likely, these mAbs bind efficiently to infectious virions.

Given that no prior human mAbs against E1 have been reported, we determined whether E1-specific mAbs had overlapping epitopes with the murine mAb CHK-166, which targets the E1 DII fusion loop [29]. The published variable domain sequences of CHK-166 [28] were cloned into the pMAZ-IgH (heavy chain) and pMAZ-IgL (light chain) plasmids that were used for expression of all DC1 and DC2-derived mAbs to generate a chimerized isotype-matched variant of CHK-166 (chCHK-166^pMAZ^) [35]. Two-phase BLI experiments in which biotinylated p62-E1 was captured on a streptavidin-coated sensor, followed by binding to chCHK-166^pMAZ^ and then binding of a human mAb while in the presence of chCHK-166^pMAZ^, were used to determine if E1 mAbs compete for binding (**Fig 2D**). DC1.56, DC2.82, and DC2.85 yielded a binding signal to the p62-E1/chCHK-166^pMAZ^ complex whereas incubation of the p62-E1/chCHK-166^pMAZ^ complex-loaded sensors in a solution containing equimolar amounts of chCHK-166^pMAZ^ resulted in no additional binding signal. When the order of binding was reversed (human mAb first, then p62-E1, then chCHK-166^pMAZ^) a similar trend was observed, with chCHK-166^pMAZ^ able to engage all human mAb/p62-E1 complexes (**S5 Fig**). Thus, our human E1 mAbs bind to epitopes that differ spatially from CHK-166.

### Neutralizing activity

**Fig 3:**
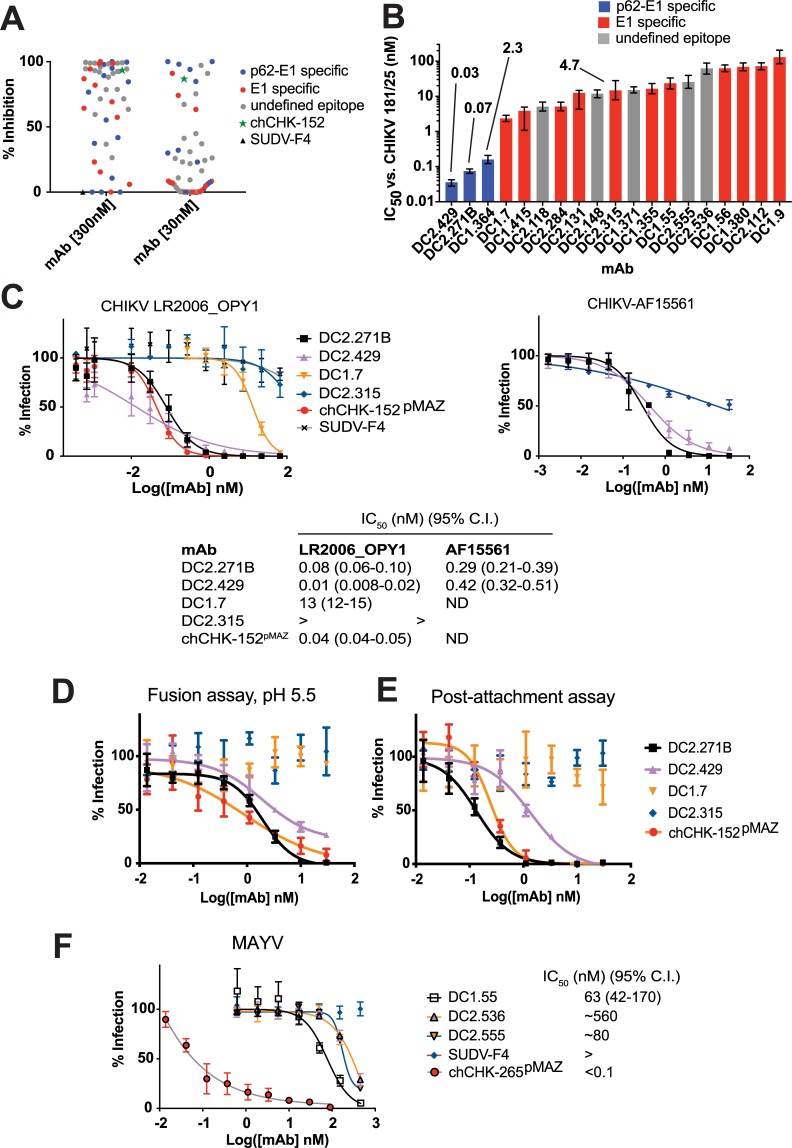
Neutralization of CHIKV by human mAbs. (**A**) Volcano plot of 46 human mAbs for their ability to inhibit infection of CHIKV 181/25 at 30 and 300 nM. (**B**) IC_50_ values for 19 of the mAbs against CHIKV 181/25. The error bars represent 95% confidence interval from data fitting, all IC_50_ values were measured twice independently with similar results. (**C**) Neutralization of ESCA genotype LR2006_OPY1 and Asian genotype AF15561 by human mAbs. (**D**) Fusion assay in which CHKV 181/25 was first bound to cells, the mAb added, and the pH lowered to pH 5.5 for 2 min at 37 ^o^C to trigger virus fusion at the plasma membrane. (**E**) Post-attachment assay, which was similar to (**D**), but following mAb addition, the virus was allowed to enter cells by endocytosis. (**F**) Cross-neutralization of MAYV by human CHIKV mAbs. A representative dataset from two experiments is shown; points represent mean ± SD.

To evaluate the capacity of the mAbs to inhibit viral infection, a focus reduction neutralization test (FRNT) using the CHIKV 181/25 vaccine strain was performed at mAb concentrations of 300 nM and 30 nM for all 46 mAbs (**Fig 3A**). At 300 nM, the majority of the mAbs could inhibit infection by >50%. These neutralizing mAbs included those binding E1 or p62-E1, and others for which the precise epitope was not identified. However, at 30 nM, only 12 mAbs showed > 50% inhibition, including some with E1, p62-E1, or undefined specificities. As a comparative control, the variable domains of murine mAb CHK-152 were expressed onto identical human constant domain background, as described above. The resulting mAb, chCHK-152^pMAZ^ strongly neutralized CHIKV infection at both 30 and 300 nM whereas the negative control mAb SUDV-F4, a Sudan virus-specific mAb with identical constant regions [36], did not.

Full dose response neutralization curves against CHIKV 181/25 were performed for 19 of the mAbs (**Fig 3B and S6 Fig**). Overall the IC_50_ values ranged from 0.03 nM to 130 nM, with those mAbs binding p62-E1 (DC2.271B, DC2.429, and DC1.364) among the most potent. A number of E1-specific mAbs (e.g., DC1.7, DC2.283, DC2.131, and DC2.315) also neutralized CHIKV 181/25 with IC_50_ values in the low/midnanomolar range (2.3–23 nM), indicating that some of the neutralization epitopes identified by our mAbs lie within the E1 subunit. The two most potent p62-E1-specific mAbs (DC2.271B and DC2.429) were tested against pathogenic CHIKV strains AF15561 (Asian genotype) and LR2006_OPY1 (East/Central/South African genotype); they potently neutralized infection of these strains with subnanomolar IC_50_ values (**Fig 3C**). chCHK-152^pMAZ^ potently neutralized CHIKV LR2006_OPY1, consistent with previous reports on fully murine, chimeric murine/human, or fully humanized CHK-152 variants [28] (**Fig 3C**). Additionally, two of the E1-specific mAbs (DC1.7 and DC2.315) were tested against CHIKV LR2006_OPY1. DC1.7 neutralized infection with an IC_50_ of 13 nM, again consistent with results using the CHIKV 181/25 vaccine strain, albeit with a higher value. In contrast, DC2.315 did not neutralize LR2006_OPY1; this mAb also was tested against AF15561 and exhibited modest and incomplete neutralization. The basis for the difference in neutralization properties for DC2.315 against CHIKV 181/25 vs. LR2006_OPY1 is unknown.

We investigated which step of CHIKV 181/25 entry was inhibited by DC2.429 and DC2.271B (p62-E1-specific), and DC1.7 and DC2.315 (E1-specific) by performing two different assays. In one assay, CHIKV 181/25 was preincubated with cells prior to mAb binding, then the pH raised to 5.5 to initiate viral membrane fusion. In this assay, mAbs DC2.429, DC2.271B as well as chCHK-152^pMAZ^ all prevented infection, but E1-specific mAbs DC1.7 and DC2.315 did not (**Fig 3D**). The second assay was similar, but following mAb addition, the cells were incubated at 37°C to allow entry. A similar trend among the p62-E1- and E1-specific mAbs was observed (**Fig 3E**). These data suggest that both DC2.429 and DC2.271B inhibit viral membrane fusion after attachment, as does chCHK152^pMAZ^ (consistent with a published study) [28]. However, the E1-specific mAbs had no effect in either assay and thus the mechanism of these mAbs remains uncertain and the subject of further investigation.

To explore the potential for cross-neutralization with other alphaviruses, 35 of the mAbs were screened for their ability to neutralize Mayaro virus (MAYV) at 300 nM and 30 nM in an FRNT (**S7 Fig**). The MAYV p62 and E1 glycoproteins are 58% and 62% identical to CHIKV p62 and E1, respectively, and previous reports have indicated that broadly neutralizing epitopes exist within domain B of E2, as typified by murine mAb CHK-265 which induces quaternary motions on the E1/E2 spike to bind an epitope consisting of residues 180–220 [25]. Of the human mAbs, only three neutralized MAYV infection (DC1.55, DC2.536, DC2.555; **Fig 3F**), albeit with lower potency than a chimerized human constant domain-matched variant of CHK-265 (chCHK-265^pMAZ^). Negative control mAb SUDV-F4 had no activity against MAYV.

### Isolation of neutralization escape viruses

**Fig 4:**
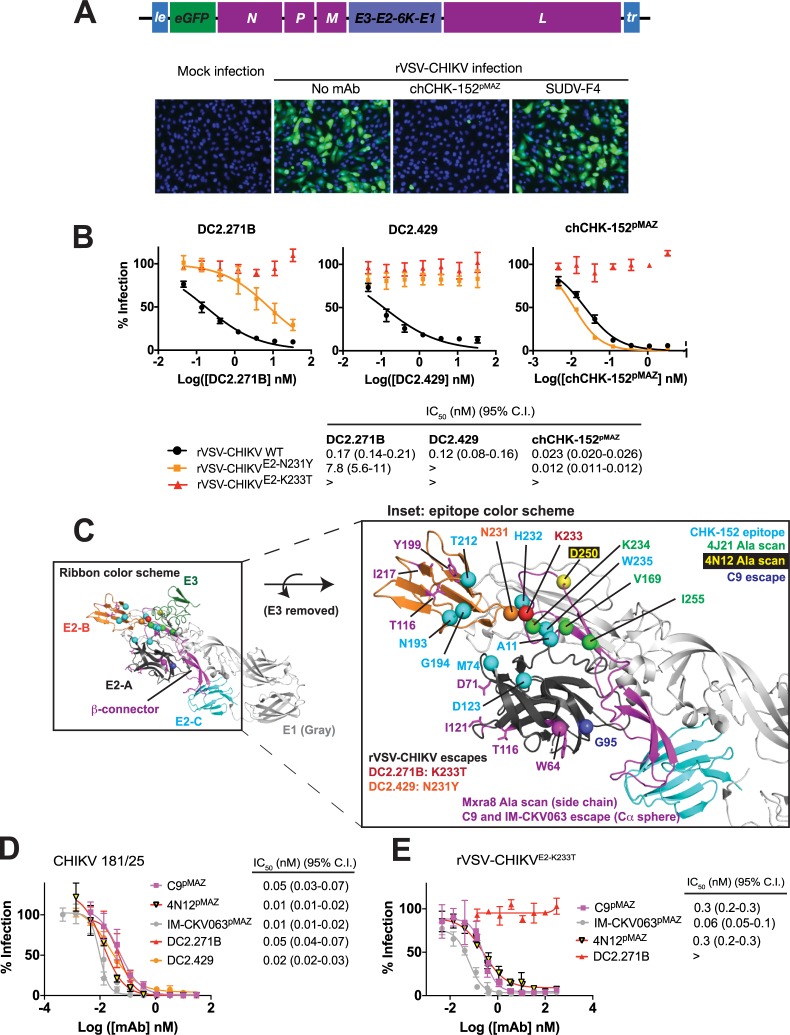
Viral escape studies for DC2.271B and DC2.429 using rVSV-CHIKV. (**A**) Schematic for the rVSV-CHIKV genome. Infection of Vero cells by rVSV-CHIKV, infection could be tracked by eGFP expression. chCHK-152^pMAZ^ inhibits this infection but SUDV-F4 does not. (**B**) Neutralization assay for rVSV-CHIKV WT and E2 viral escape mutations by DC2.271B, DC2.429, and chCHK-152^pMAZ^. Data are pooled from two experiments, each performed in duplicate, (points represent mean ± SD). (**C**) Location of rVSV-CHIKV escape mutations for DC2.429 and DC2.271B (orange and red Cα spheres, respectively) mapped onto the p62-E1 X-ray structure. E2 domains as well as E3 are colored as in **Fig 1A**; E1 is colored gray. Also shown are previously reported alanine scanning or viral escape mutations that ablate binding for 4N12 (parent of SVIR001, yellow Cα sphere), 4J21 (green Cα spheres), C9 (magenta Cα sphere), and IMCV-063 (blue Cα sphere). The structural epitope for murine mAb CHK-152, as mapped by cryo-EM is shown as cyan Cα spheres. The alanine scanning mutations that reduced Mxra8 binding are shown as magenta side chains. (**D**) Comparison of neutralizing potency for DC2.271B, DC2.429, C9^pMAZ^, IM-CKV063^pMAZ^, and 4N12^pMAZ^. (**E**) Neutralization of DC2.271B viral escape mutant rVSV-CHIKV^E2-K233T^ by C9^pMAZ^, IM-CKV063^pMAZ^, and 4N12^pMAZ^. For panels **B** and **C**, data are pooled from two experiments, each performed in duplicate (points represent mean ± SD). Panel **D**, two independent experiments were performed in triplicate with similar results; a representative dataset is shown.

To map the potential epitopes of the two most potent p62-E1-specific mAbs (DC2.271B and DC2.429) as well as two of the E1-specific mAbs (DC1.7 and DC2.315), we generated a replication-competent vesicular stomatitis virus clone bearing CHIKV E3-E2-6K-E1 genes in place of the native glycoprotein G (rVSV-CHIKV) (**Fig 4A**). Similar viruses have been generated and evaluated as potential vaccine candidates for CHIKV, ebolaviruses, and arenaviruses [37–39]. The rVSV-CHIKV particle encodes an enhanced green fluorescent protein (eGFP) as an additional transcription unit that allows quantification of infection of Vero cells by automated fluorescence microscopy (**Fig 4A**). rVSV-CHIKV was neutralized by chCHK-152^pMAZ^ but not by SUDV-F4 (**Fig 4A and 4B and S8A Fig**). Despite the different morphologies of VSV and CHIKV particles (bullet-shaped and spheroid, respectively) [11, 40], the observation that rVSV-CHIKV is efficiently neutralized by chCHK-152^pMAZ^ suggest that it recapitulates the critical features of CHIKV entry.

We found that DC2.271B and DC2.429 efficiently neutralized rVSV-CHIKV (**Fig 4B and S8B Fig**). rVSV-CHIKV was serially passaged against each mAb, and individual plaques from the resulting escape populations were isolated, sequenced, and characterized. For DC2.271B and DC2.429, single escape mutations of E2 K233T or E2 N231Y were isolated, respectively (**Fig 4B and 4C**). Both of these residues are located at the junction between the β-connector and B domains of E2 and just outside the region that is occluded by E3 on the p62-E1 hybrid protein. The epitopes for previously reported human neutralizing mAbs against CHIKV E2 (1H12, 8I4, 4J21, and 3N23) include an adjacent residue, K234, as mapped by an alanine scanning library of cell-surface expressed E1/E2 (the 3N23 epitope also includes K233) (**Fig 4C**) [10, 31]. Furthermore, the cryo-electron microscopy (cryo-EM)-mapped structural epitope of CHK-152 includes residues in this region (H232 and W235) as well as residues on the B domain [11]. The epitope of the non-neutralizing human mAb 1M9 includes N231, although DC2.429 is potently neutralizing while likely engaging a similar epitope as 1M9. The viral escape mutations for other human neutralizing mAbs 4N12 (parent of SVIR001), C9, and IM-CKV063 were located on distal regions of the E2 subunit [27, 41]. Together, these results indicate that the β-connector and B domain regions constitute epitopes recognized by a number of anti-CHIKV neutralizing antibodies. This region is distal to the putative Mxra8 receptor high affinity site, which includes major contact residues in the A and B domains (**Fig 4C**) [16, 17].

The viral escape mutations for DC2.271B and DC2.429 on rVSV-CHIKV are proximal to one another and lie in the middle of the cluster of residues identified as the structural epitope of CHK-152 by cryo-EM studies (**Fig 4C**), suggesting that some or all of the epitopes for these three antibodies may be shared. To further investigate this hypothesis, all three mAbs were tested against both the rVSV-CHIKV^E2-K233T^ (DC2.271B viral escape) and the rVSV-CHIKVK^E2-N231Y^ (DC2.429 viral escape) (**Fig 4C**). chCHK-152^pMAZ^ neutralized rVSV-CHIKV^E2-N231Y^ with similar potency as WT rVSV-CHIKV but did not neutralize rVSV-CHIKV^E2-K233T^. DC2.429 did not neutralize either viral escape mutant, and DC2.271B weakly neutralized rVSV-CHIKV^E2-N231Y^. These data suggest that the DC2.271B epitope includes residues for binding both DC2.429 and CHK-152, as neither of these mAbs could neutralize the rVSV-CHIKV^E2-K233T^ viral escape mutant that was selected against DC2.271B. In contrast, the epitope for DC2.429 may be only partially shared with DC2.271B and may not be shared at all with CHK-152, since the DC2.429 viral escape mutant (rVSV-CHIKV^E2-N231Y^) was fully neutralized by chCHK-152^pMAZ^ and partially neutralized by DC2.271B. However, these studies do not rule out the possibility that the viral escape mutations described here induce conformational effects, rather than directly ablating binding interactions. Furthermore, these data do not unequivocally demonstrate competition of the three mAbs. Competition ELISA and BLI experiments with DC2.271B, DC2.429, and chCHK-152^pMAZ^ did not yield interpretable results, likely due to the weak binding of the human mAbs toward the monomeric p62-E1 hybrid protein (above).

To compare neutralizing activity of DC2.271B and DC2.429 with the most efficacious of the previously reported human mAbs [30, 31], we generated versions of C9, IM-CKV063 and 4N12 (parent of SINV001) expressed from the pMAZ platform (C9^pMAZ^, IM-CKV063^pMAZ^, and 4N12^pMAZ^) and assessed their capacity to neutralize CHIKV 181/25 infection. We found that C9^pMAZ^, 4N12^pMAZ^ and IM-CKV063^pMAZ^ neutralized CHIKV 181/25 similarly to DC2.271B and DC2.429 (**Fig 4D**). To further examine the degree to which the DC2.271B epitope overlapped with these three human mAbs, they were tested for their capacity to neutralize rVSV-CHIKV^E2-K233T^ (DC2.271B viral escape mutant, **Fig 4E**). C9^pMAZ^, 4N12^pMAZ^, and IM-CKV063 potently neutralized rVSV-CHIKV^E2-K233T^. These results indicate that the DC2.271B epitope is distinct from those of previously reported human mAbs.

**Fig 5:**
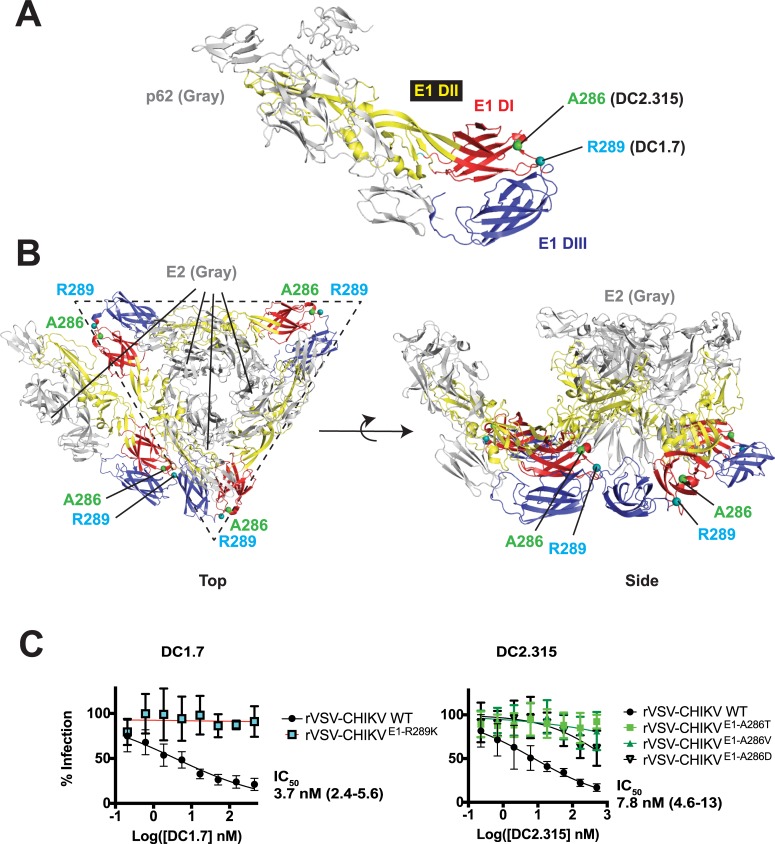
Viral escape studies with E1-targeting mAbs. Location of A286 and R289 on the p62-E1 X-ray structure (**A**, PDB ID: 3N40) [9] or on the E1/E2 cryoEM heterohexamer (**B**, PDB ID: 3J2W) [11]. For clarity, p62 or E2 subunits are colored gray whereas E1 domains DI, DII, and DIII colored as per **Fig 1**. In panel B, a complete prefusion E1/E2 hexameric spike (outlined with dotted line) is illustrated, along with an E1/E2 heterodimer from an adjacent spike, to depict relative orientation within adjacent spikes. (**C**) Neutralization studies with WT VSV-CHIKV and viral escape mutants. Data are pooled from two experiments, each performed in duplicate or triplicate (points represent mean ± SD).

A similar viral escape study was performed with rVSV-CHIKV and E1-specific mAbs D1.7 and DC2.315 (**Fig 5**). For E1-specific mAb DC1.7, a single escape mutation of E1 R289K was isolated, which lies in the linker region leading into domain III (DIII) (**Fig 5A and 5B**). For DC2.315, three independent escape mutants at position E1 A286 were identified (A286T, A286V, and A286D, **Fig 5C**). This residue is located at the border of domain I (DI), immediately preceding the linker that leads into DIII (**Fig 5A**). When considered within the context of the E1/E2 prefusion hexamer, the escape mutations for both DC1.7 and DC2.315 lie near the edge of the triangular spike (**Fig 5B**). Topologically, one potential mechanism by which these mAbs could access this site is by engaging the underlying E1 subunit in between adjacent E2 subunits (see **Fig 5B**, side view). The observation that this region is targeted by neutralizing mAbs, albeit strain-dependently and with modest potency, is notable since DIII is the most structurally mobile region of E1 during its conformational rearrangements to mediate viral membrane fusion [42, 43]. However, the mechanism of neutralization for DC1.7 and DC2.315 is likely more complex as these mAbs did not inhibit viral membrane fusion at the plasma membrane (**Fig 3D**). The relatively modest potency as well as the fact the DC1.7 and DC2.315 neutralization varies per strain suggests that this epitope is not a site of universal susceptibility. An alignment of this region from several CHIKV E1 sequences reveals it is conserved (**S9 Fig**), particularly at positions A286 and R289, and thus the variable neutralizing capacity among strains is not due to sequence differences.

### Protective capacity of mAbs in mice

Four mAbs (DC2.271B, DC2.429, DC1.7, and DC2.315) were tested for their ability to protect mice from a highly stringent, lethal viral challenge with CHIKV LR2006_OPY1, using 3-week old C57BL/6 mice rendered immunodeficient by treatment with the anti-Ifnar1 mAb MAR1-5A3 [44]. The CHIKV mAbs (100 μg, ~6 mg/kg) were administered one day prior to virus infection. DC2.271B and DC2.429 were the most potently neutralizing among the E2 mAbs. Although neutralization by DC1.7 was relatively modest and DC2.315 was strain-dependent (non-neutralizing against CHIKV LR2006_OPY1), we nonetheless determined if mAbs binding E1 in this region could afford protection. In other pathogens, such as ebolaviruses, some non-neutralizing mAbs protect *in vivo* [45]. Human mAbs targeting E1 DI, DIII, or the DI-DIII linker have not previously been studied for protection against CHIKV *in vivo*.

**Fig 6:**
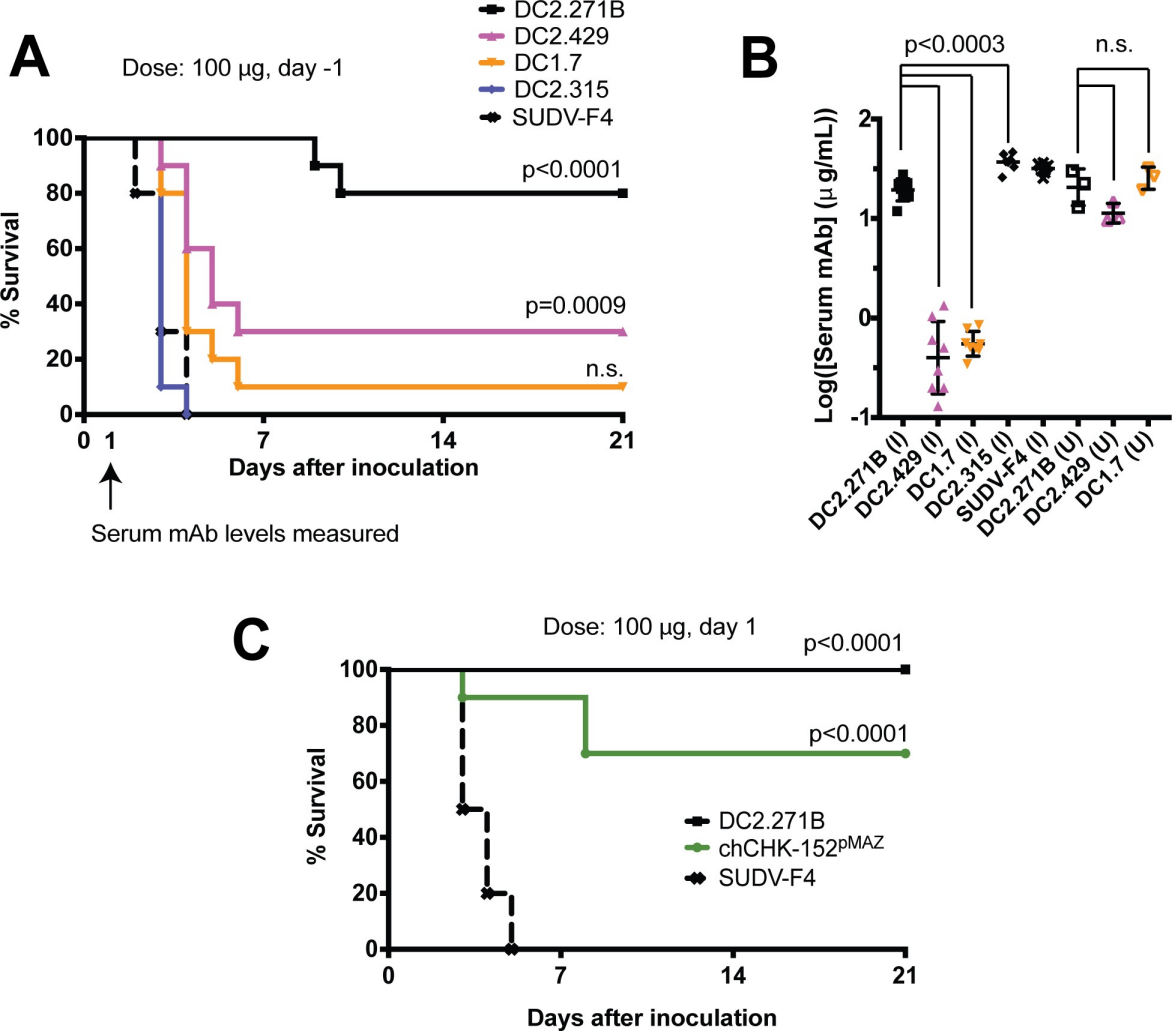
*In vivo* properties of human mAbs in mice. (**A**) Protective efficacy using a prophylactic dosing regime (day -1) against CHIKV LR2006_OPY1 in 3-week old mice rendered immunodeficient with anti-Ifnar1 mAb. Survival curves were compared using the log-rank test with a Bonferroni correction. Results were combined from two independent experiments of five mice per treatment group (n = 10). (B) Serum mAb levels 48 hours after mAb administration in infected mice from (A) (“I”, closed symbols) and 60 hours after mAb administration in uninfected mice (“U”, open symbols). There were three mice per group points represent mean ± SD). Serum mAb levels were compared by unpaired t-test. (**C**) Therapeutic dosing regimen, similar to (**A**) but CHIKV mAbs were administered at day +1. Analysis as in (**A**).

All mice receiving the SUDV-F4 negative control mAb succumbed to infection within four days. In contrast, 80% of mice receiving DC2.271B survived the challenge. mAb DC2.429 afforded a lesser but significant survival advantage (30%). Neither DC1.7 nor DC2.315 conferred significant *in vivo* protection from CHIKV infection (**Fig 6A**). To gain insight into factors contributing to *in vivo* efficacy, the serum mAb levels during the challenge were measured by sampling 2 days after IP administration of the mAb and 1 day after infection (**Fig 6B**). DC2.271B, DC2.315, and negative control mAb SUDV-F4 were present at average (across mice) serum concentrations of 20 ± 2 μg/mL, 38 ± 3 μg/mL and 32 ± 1 μg/mL, respectively, whereas DC2.429 and DC1.7 were present at >30-fold lower serum concentrations (0.5 ± 0.1 and 0.6 ± 0.1 μg/mL, respectively). Early clearance of mAb could be due to the presence of aggregates. To explore this possibility, mAbs were subjected to SEC-HPLC analysis and a small (n = 3) pharmacokinetic study in uninfected mice. All of the DC1.7, DC2.315, and SUDV-F4 were found to be > 98% monomeric (**S10 Fig**). The serum levels of DC2.271B, DC2.429, and DC1.7 were found to be 26 ± 4, 22 ± 5, and 12 ± 2 μg/mL (average across three mice) when administered at protective doses (**Fig 6B**). Together, these results indicate that DC2.429 and DC1.7 are cleared from serum more rapidly during the course of CHIKV infection and treatment than DC2.271B and DC2.315, but that this early clearance is not due to aggregates and depends on the presence of virus. Perhaps related to this effect, the half-life of HIV-1 bNAbs was found to be reduced in human clinical trials (~ 3 days) when used therapeutically for patients coming off antiretroviral therapy [46]. A possible mechanism for this observation is that mAbs bind virus in the bloodstream, and the antibody-virus complex is cleared. However, why this affects DC2.429 and DC1.7 but not DC2.271B or DC2.315 warrants further investigation.

To further examine the therapeutic potential of DC2.271B, a second study was performed in which 100 μg of DC2.271B, SUDV-F4, or chCHK-152^pMAZ^ (for comparison) was administered one day after viral challenge (**Fig 6C**). DC2.271B afforded 100% protection under this dosing regimen; chCHK-152^pMAZ^, which was fully protective in adult *Ifnar*^-/-^ mice [28], was found to be 70% protective, whereas all mice treated with SUDV-F4 succumbed to infection by the 5^th^ day. These results illustrate the therapeutic potential of DC2.271B.

### Recognition requirements of DC2.271B

**Fig 7:**
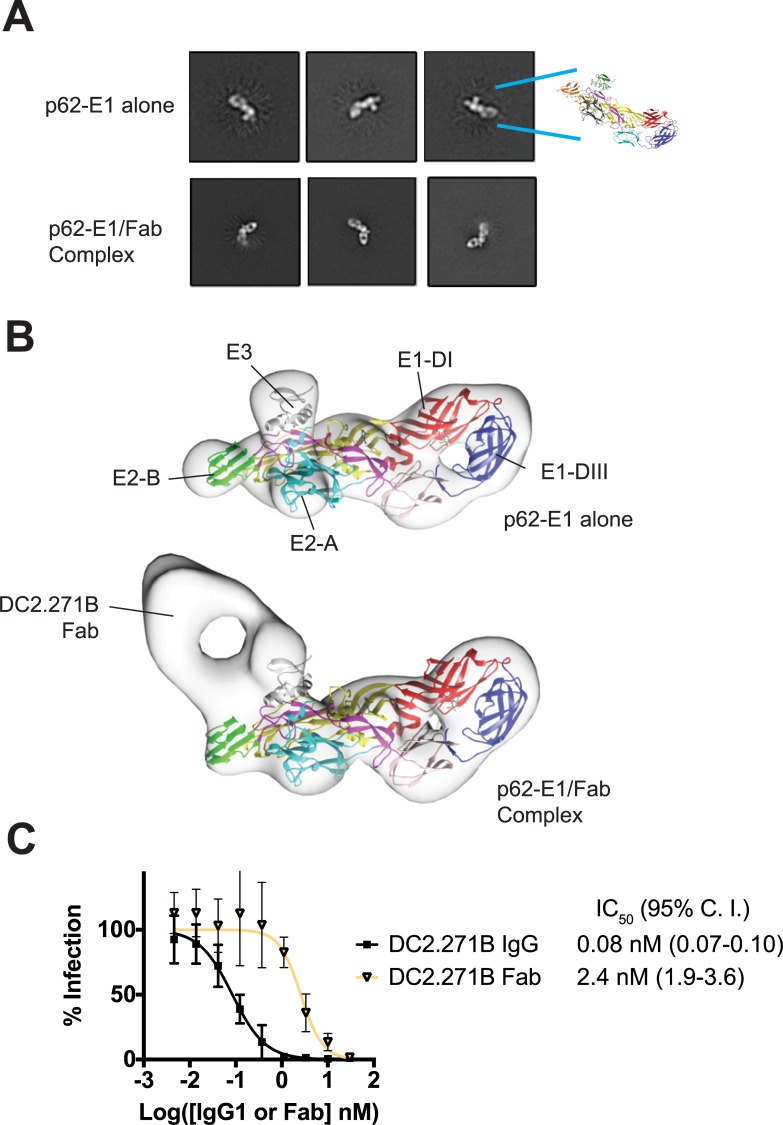
Recognition requirements for DC2.271B. CryoEM visualization (**A)** and single particle three-dimensional reconstruction (**B**) of p62-E1 alone and in complex with DC2.271B Fab. (**C**) Neutralization of CHIKV 181/25 by DC2.271B IgG1 and Fab. Data are pooled from two experiments each performed in triplicate (points represent mean ± SD).

Given the highly protective properties of DC2.271B, we explored the recognition requirements of this mAb in the context of the isolated glycoprotein and viral particle. The antigen-binding fragment (Fab) of DC2.271B was generated by papain digestion, and then the complex of Fab with p62-E1 was purified. Both the p62-E1 hybrid protein alone as well as the p62-E1/DC2.271B complex (1:1) were visualized by negative stain electron microscopy (**Fig 7A and 7B**), with the twisted β-plate architecture of the p62-E1 evident as well as the characteristic “hole” between the heavy and light chains of the DC2.271B Fab. Three-dimensional reconstruction from the p62-E1 and p62-E1/DC2.271B revealed that the Fab density appears offset to E3, near the B domain (**Fig 7B**). This structural model is consistent with the location of the viral escape mutation (VSV-CHIKV^E2-K233T^). Together, these data indicate that the epitope for DC2.271B lies in the loop region between the β-connector and the B domain. This structural epitope is proximal to, but not occluded by E3, and in the reconstruction of the Fab/p62-E1 complex, density corresponding to what is presumably E3 abuts the Fab. Notably, viral escape rVSV-CHIKV^E2-K233T^ is proximal to E3 yet E3 does not sterically block binding of DC2.271B. To compare the effects of bivalent binding vs. monovalent binding, the DC2.271B Fab was tested for neutralization against CHIKV 181/25 and found to have neutralizing activity, but showed a 30-fold reduced potency relative to the intact IgG (**Fig 7C**). Thus, bivalent crosslinking of epitopes from adjacent subunits is not a requirement for neutralization by DC2.271B.

## Discussion

We describe the characterization of a panel of human mAbs against CHIKV isolated by single B cell sorting. Human mAbs against CHIKV have been isolated previously by phage display and hybridoma methods [31, 47]. Here, human mAbs targeting both E1 and E2 subunits were isolated, in contrast to previously reported hybridoma-derived human mAbs that targeted exclusively E2 [31]. Previously described murine E1 mAbs (*e*.*g*., CHK-166) were identified by immunization with virus [28], and the phage-derived human mAbs IM-CKV061, 062, and 063 contain epitopes that span both E1 and E2 [47]. In contrast, we show here that E1 mAbs DC1.7 and DC2.315 bind exclusively to the E1 subunit, and neutralization escape mutants suggest that both of these mAbs target epitopes near E1 DIII. IM-CKV063 as well as CHK-166 provide protection from lethal challenge in different mouse models and bind near the fusion loop of DI. In contrast, DC1.7 and DC2.315 exhibited lower neutralization potential relative to E2-targeting mAbs and did not confer protection in 3-week old mice rendered immunodeficient with an anti-Ifnar1 mAb.

For DC1.7, the lack of protection may correlate to some degree with the lower mAb levels in infected mice at day 2, whereas DC2.315 was present at levels similar to those of the protective DC2.271B. Although DIII is a common target for neutralization in flavivirus E, the analogous structural domain on alphavirus E1 is likely occluded by E2, which comprises the majority of the exposed surface in cryoEM structures of intact CHIKV VLPs [11]. Whatever neutralizing activity that DC1.7 and DC2.315 contain may result from their ability to engage their epitopes between E2 subunits (**Fig 5B** side) or during viral breathing [48]. However, this neutralizing activity was not sufficient to protect *in vivo* at least in the case of DC2.315. Our BLI analysis suggests that other E1 mAbs in the panel do not compete with CHK-166, and thus it remains to be determined whether mAbs to other epitopes in E1 have protective capacity. The differences in epitopes targeted by human mAbs may reflect differences in individual responses, but also may reflect differences in isolation and selection methods. In our case, the p62-E1, which contains a partially exposed E1, was used for the antigen sorting whereas other methods relied on screening against whole virions or VLPs. While E1 epitopes are largely sheltered from mAb binding on the viral particle, it is nonetheless possible that such regions elicit immune response because E1 or segments of E1 (or other CHIKV proteins) may be secreted by infected cells, be exposed on the surface of infected cells prior to budding, or presented by immune cells following proteolysis.

Viral escape mutations to DC2.271B and DC2.429 in rVSV-CHIKV were adjacent to one another on the region between the β-connector and the A domain, and thus the mAbs likely bind in similar fashion. However, DC2.271B conferred a strong survival advantage (>80%) *in vivo*, whereas the protective properties of DC2.429 were lower (30%). This difference in protection is most likely due to differences in the serum mAb concentrations in infected mice, as DC2.271B was present at 37-fold higher serum concentration than DC2.429 (20 vs. 0.5 μg/mL) in the infected mice two days after mAb dosing and one day after viral challenge. The low serum concentration of DC2.429 is not due to antibody aggregation, and was dependent on presence of virus, as uninfected mice maintained much higher levels of serum DC2.429 (22 μg/mL) three days after mAb administration. The basis for the difference in serum mAb levels during the challenge between DC2.271B, which was highly protective, and DC2.429, which was moderately protective, is not clear, since both mAbs neutralize virus with similar potency, bind in similar regions, and have the same isotype. Nonetheless, the results suggest that intrinsic *in vivo* properties of otherwise similar mAbs can contribute to their overall protective efficacy. A similar contrast in serum mAb levels was seen with the E1-binding mAbs DC1.7 and DC2.315, but neither had any protective properties.

Protection studies with DC2.271B confirmed the region near the β-connector and B domain as a general site of susceptibility for neutralization and, in some cases, *in vivo* protection. This region is also targeted by protective mAbs 4J21 (human), 5M16 (human), 4N12 (human, the parent of SVIR001) and CHK-152 (mouse) all of which were shown protective in adult *Ifnar*^-/-^ mice [28, 31]. SVIR001 was further shown to decrease viremia in joint tissues in nonhuman primates, and the parent of SVIR001 (4N12) was equivalent in neutralization potency *in vitro* to DC2.271B and DC2.429 against CHIKV 181/25 [24]. The high affinity binding region of Mxra8, an entry receptor for arthritogenic alphaviruses, lies across the A- and -B domains of adjacent E2 subunits in the trimer [15–17]. This Mxra8 binding region is distal to epitopes of β-connector/B domain mAbs; nonetheless, 4J21 was shown to compete with an Mxra8 ectodomain-Fc fusion protein for binding to the CHIKV virion[15]. Although we have not tested whether DC2.271B similarly interferes with Mxra8 binding, based on analogy to 4J21 and 5M16, it is possible that it blocks binding to Mxra8 which might contribute to its neutralizing activity. The viral escape mutations of both DC2.271B and DC2.429 also were distal to epitopes of C9 and IM-CKV063 (A domain) as inferred by alanine scanning mutagenesis or viral escape. Moreover, the DC2.271B viral escape mutant rVSV-CHIKV^K233T^ was susceptible to our own recombinant preparations of these mAbs, as well as to 4N12^pMAZ^ [30, 41]. Recent work has shown that the neutralizing mAbs C9 and IM-CKV063 also can crosslink CHIKV envelope proteins at the infected cell surface and inhibit viral egress [41]. The action of several protective mAbs targeting distinct epitopes, or with distinct mechanisms of action, could be combined for synergistic protective effect.

Protective mAbs have potential for use as immunotherapies against viral diseases. Although most human CHIKV infections are not lethal, the virus is widespread and can be associated with a painful arthralgia and arthritis that persists for months or years. Protective CHIKV mAbs could be used to ameliorate musculoskeletal disease, although this therapeutic modality would likely require mAbs having the capacity to clear virus from joint tissues [49]. Alternatively, protective mAbs could be used as prophylaxis in CHIKV endemic regions. With appropriate Fc engineering modifications, mAb half-life can be extended to months which could allow short-term protection for individuals traveling to endemic regions [23, 50]. The results presented here suggest that DC2.271B, and perhaps other mAbs in the panel, could serve as such immunotherapies or prophylactic candidates. Further testing in larger animals will be required to fully assess this potential.

## Materials and methods

### Ethics statement

To study naturally acquired antibodies to CHIKV, we recruited healthy adult patients who had a history of symptomatic CHIKV infection. Patients were identified either through the Montefiore Medical Center Microbiology laboratory with a positive CHIKV serology or from the community with a self-reported diagnosis of CHIKV. After written informed consent, details of their CHIKV illness was recorded and blood samples (~40 mL per sample) were collected. The study protocol was approved by the Institutional Review Board of the Albert Einstein College of Medicine (protocol IRB# 2016–6137). CHIKV immune status was confirmed by serum ELISA.

This mouse CHIKV challenge study was carried out in accordance with the recommendations in the Guide for the Care and Use of Laboratory Animals of the National Institutes of Health. The protocols were approved by the Institutional Animal Care and Use Committee at the Washington University School of Medicine (Assurance number A3381-01) under animal use approval number 20180234.

### CHIKV p62-E1 and E1' production

The CHIKV-115 p62-E1 construct was a gift from Dr. Félix Rey (Institut Pasteur), and the recombinant protein was purified from S2 cells as previously described [9]. The construct contained the p62 and E1 ectodomains joined by a glycine-serine linker with a double strep-tag at the C-terminus (IBA Lifesciences). The p62 furin cleavage site (between E2 and E3) was mutated to prevent furin cleavage [9]. E1' was expressed in S2 cells and purified as above and as previously described [42].

### Viruses

CHIKV 181/25 was obtained from Dr. Robert B. Tesh (University of Texas Medical Branch). The Mayaro Guyane virus (NR-49911) was obtained through BEI Resources, NIAID, NIH, as part of the World Reference Center for Emerging Viruses and Arboviruses (WRCEVA) program. CHIKV 181/25 and MAYV Guyane viruses were propagated and titered on BHK-21 cells (ATCC). CHIKV LR2006_OPY1 and AF15561 (United States Army Medical Research Institute of Infectious Diseases) were used as previously described [28, 29].

### Isolation of PBMCs

Forty mL of whole blood was collected from patients using K_2_EDTA blood collection tubes (BD Vacutainer, Franklin Lakes, New Jersey). Plasma (~15 mL) was separated, aliquoted and frozen. To isolate PBMCs using a density gradient separation, blood was mixed with 1:1 ratio of Hanks Balanced Salt Solution (HBSS) and layered over equal volume of Ficoll-Paque (GE: 17-5442-02) and centrifuged per the manufacturer’s protocol. The PBMC layer was collected, washed with HBSS, centrifuged at 400 x g, and frozen at 4x10^6^ cells/mL in heat-inactivated FBS (Gibco) and 5% DMSO and then stored in liquid nitrogen.

### Isolation of CHIKV mAbs by single B cell sorting

Approximately 8x10^6^ cells/mL were stained using anti-human CD8 (PE-Cy7), CD3(PE-Cy7), CD14(PE-Cy7), CD20 (PB), CD27 (APC), IgG (FITC), and biotinylated p62-E1 hybrid protein. p62-E1 was biotinylated using EZ-Link Sulfo-NHS-LC-Biotin (Life Technologies) followed by buffer exchange using Amicon 30,000 MW cut-off spin columns (Millipore) into PBS pH 7.4. Biotinylated p62-E1 was used at a concentration of 100 nM and detected using streptavidin-PE (Invitrogen) at 1:500 dilution. Single B cells were sorted into 8-strip PCR tubes (USA scientific) containing 4μl/well of lysis buffer [RNasin Ribonuclease Inhibitors (Promega) 2U/well, 0.005 M DTT (Invitrogen), PBS, nuclease free H_2_O] using FACS Aria high-speed cell sorter flow cytometer (Becton Dickinson). Tubes were frozen on dry ice and stored at stored at -80°C.

IgH and Igκ variable gene transcripts were amplified using an RT-PCR and two-step nested PCR strategy. A primer set specific to IgG leader sequences, constant regions and V-region heavy/light chain families was used for antibody variable region recovery [33]. The second round PCR primer set had 35 base pairs of 5' and 3' homology to the heavy and light chain expression vectors pMAZ-IgH and pMAZ-IgL [35]. Gibson cloning reactions were performed using 100 ng of purified PCR and 50 ng of cut heavy and light chain plasmids containing IgG_1_ constant-region framework. Chemical transformations were done using 10 μl of DH5-α (New England BioLabs) and 1 μl of Gibson reaction mix. Individual colonies were picked and sequenced for downstream analysis and characterization.

### Expression and purification of mAbs and Fab fragments

Sequences for all antibodies were cloned into pMAZ-IgL and pMAZ-IgH for recombinant expression [35]. Antibodies used for binding and neutralization screens were expressed in FreeStyle 293-F cells (ThermoFisher) by transient co-transfection of 1:1 ratio of heavy and light chain plasmids. Cells were passaged to 5.0 × 10^5^ cells per ml. A transfection mixture of DNA diluted in PBS (0.67 μg total plasmid DNA per ml of culture) was prepared on day of transfection. Addition of transfection agent Polyethylieneimine “MAX” (PEI) (Polysciences Inc) at a DNA-to-PEI ratio of 1:3 to diluted DNA and incubated at room temperature for 15 min. The transfection mixture was then added to culture via drop-wise addition. At six days post-transfection, cultures were harvested by centrifugation at 4,000 x g for 15 min, and incubated with Protein A agarose (Thermo Scientific) at 4°C for 90 min. Protein A resin containing bound mAbs was then passed through a protein purification column (BioRad) and washed twice with Pierce Gentle Ag/Ab Binding Buffer, pH 8.0 (Thermo Scientific). Antibodies were eluted Pierce Gentle Ag/Ab Elution Buffer, pH 6.6 (Thermo Scientific) and desalted into 150 mM HEPES, 200 mM NaCl, pH 7.4 using PD-10 Desalting Columns (GE Healthcare). Fab fragments were generating by digestion of IgG1 using Pierce Fab preparation kit (Thermo Scientific) as per manufacturers protocol. Briefly IgG was incubated with papain for 4 h at 37°C, and the Fab and the Fc mixtures were passed over Protein A agarose to remove Fc fragments and undigested Fc. Fab fraction was then buffer exchanged into 150 mM HEPES, 200 mM NaCl, pH 7.4.

### Immunoprecipitation of viral proteins from infected cells

BHK-21 cells were cultured at 37˚C in complete media (Dulbecco’s modified Eagle’s medium (DMEM) with 5% fetal bovine serum, 10% tryptose phosphate broth, 100 U penicillin/mL, and 100 ug streptomycin/ml) and seeded 24 h prior to infection. Cells were inoculated with CHIKV 181/25 at 10 PFU/cell for 4 h, washed three times, and placed back into complete media. At 8 h post-infection, cells were washed once with minimal essential media (MEM) lacking cysteine and methionine and then labeled with 50 uCi/mL of [^35^S]methionine/cysteine for 2 h. The cells were washed three times with ice-cold PBS before solubilizing on ice with lysis buffer (50 mM Tris-Cl pH 7.4, 100 mM NaCl, 1% Triton X-100, 1 mM EDTA, and one complete protease inhibitor tablet/10 ml (Roche)). Cell debris was removed by centrifugation at 20,000 x g 4˚C 10 min. The soluble lysate was aliquoted and frozen at -80˚C. Approximately 1 μg of each candidate antibody was incubated with an individual lysate aliquot for 1 h in the presence of 0.1% SDS and the immunoprecipitate was retrieved with Protein A agarose (Pierce) for 3 h at 4˚C. The beads were washed four times with RIPA buffer and once with PBS. The samples were then boiled in SDS sample buffer supplemented with dithiothreitol, alkylated with iodoacetamide at 37˚C, and analyzed by SDS-PAGE and fluorography.

### Biolayer interferometry (BLI)

IgG binding to p62-E1 and E1' was determined by BLI measurements using OctetRed system (ForteBio, Pall LLC). For single-phase binding experiments, global data fitting to a 1:1 binding model was used to estimate values for the k_on_ (association rate constant), k_off_ (dissociation rate constant), and K_D_ (equilibrium dissociation constant). IgGs were immobilized on anti-human Fc capture sensors (Pall Life sciences). Data were analyzed using ForteBio Data Analysis Software 9. For double phase binning experiments, biotinylated p62-E1 was first bound to streptavidin-coated sensor, and then the first mAb bound to saturation. The sensor was then transferred to a second well containing equimolar amounts of the first and competing mAbs.

### p62-E1 and E1’ ELISA

Initial antibody binding screening against p62-E1 was performed by coating 250 ng/well diluted in PBS in half-area 96-well high binding plates (Costar). Wells were blocked with 3% BSA at 37°C for 2 h. Antibody dilutions at 300 nM and 30 nM were performed in PB-T (PBS pH 7.4, 0.5% BSA, 0.05% Tween) and incubated 1 h at 37°C. After antibody binding plates were washed with PBS-T (PBS pH 7.4, 0.005% Tween-20) five times. Horseradish peroxidase conjugated-(HRP)-Protein A (life technologies) diluted at 1:2000 in PB-T was added in for 1 h at 37°C. Plates were washed five times with PBS-T and developed using TMB (Thermo Fischer). Optical density at 450 nm was read on Synergy H4 Hybrid reader (BioTek). Procedures were similar for full (8-point) ELISA curves and serum ELISA, except that initial stock of mAb or serum were serially diluted. Experiments with E1’ as the target were similar.

### Focus reduction neutralization test with CHIKV 181/25

Serial dilution of mAbs were incubated with 100–150 FFU of CHIKV 181–25 vaccine strain for 1 h at 37°C. Antibody-virus complexes were then added to Vero cell monolayers in 96-well plates. Infection proceeded for 90 min at 37°C and cells were then overlaid with 0.5% carboxylmethylcellulose in Modified Eagle Media (MEM), supplemented with heat inactivated 2% FBS and 10 mM Hepes pH 7.4. Plates were fixed 16 h post-infection with 1% PFA diluted in PBS. After fixation, plates were incubated with 250 ng/mL of 5G11 (USAMRIID) and horseradish peroxidase (HRP)-conjugated goat anti-mouse IgG in PBS supplemented with 0.1% Saponin and 0.1% BSA. Foci were then visualized using TrueBlue Peroxidase substrate (KPL). Developed foci where quantified on ImmunoSpot S6 Macroanalyzer (Cellular Technologies Ltd.). Infection in wells containing mAb was calculated relative to wells containing CHIKV 181/25 alone. Non-linear regression analysis was performed using Prism 7 software (GraphPad Software, La Jolla CA).

### CHIKV-AF15561 microneutralization assay

Serial dilutions of mAbs were prepared in infection media (2% FBS MEM) and incubated with CHIKV-AF15561 virus for 1 h at 37°C. Vero E6 cells (ATCC) then were exposed to antibody/virus inoculum at an MOI of 1.5 plaque-forming units (PFUs)/cell for 1 h at 37°C before it was removed and replaced with fresh culture media (5% FBS MEM). At 24 h post-infection, cells were fixed in 10% formalin for 24 h prior to removal from containment. Cells were permeabilized with 0.2% Triton X-100 (Sigma-Aldrich) for 10 min, blocked and incubated with 2 μg/ml CHIKV-specific 5G11 for 1 h at RT. Cells were washed with PBS, incubated with anti-mouse IgG conjugated to Alexa488 (Sigma-Aldrich), washed again and counterstained with Hoechst stain (Invitrogen). Infection was quantitated by automated fluorescence microscopy, as described [51].

### Focus reduction neutralization test with CHIKV LR2006_OPY1

Focus reduction neutralization tests (FRNT) were performed as previously described [28]. Briefly, serial dilutions of mAb were incubated with 100 FFU of CHIKV LR2006_OPY1 for 1 h at 37°C. MAb-virus complexes were added to Vero cells (ATCC) in 96-well plates. After 1 h, cells were overlaid with 1% (w/v) methylcellulose in Modified Eagle Media (MEM) supplemented with 4% FBS. Plates were fixed with 1% PFA in PBS 18 h later. Plates were incubated sequentially with 500 ng/ml of mouse anti-CHK-11 [28] and horseradish peroxidase (HRP)-conjugated goat anti-mouse IgG in PBS supplemented with 0.1% saponin and 0.1% BSA. CHIKV LR2006_OPY1 foci were visualized using TrueBlue peroxidase substrate (KPL) and quantitated on an ImmunoSpot macroanalyzer (Cellular Technologies Ltd). The IC_50_ was calculated using non-linear regression analysis constraining the bottom to 0 and top to 100 after comparison to wells infected with CHIKV-LR in the absence of antibody.

### Generation of recombinant vesicular stomatitis virus (rVSVs) bearing CHIKV glycoproteins (rVSV-CHIKV)

Human codon optimized sequence of the CHIKV E3-E2-6K-E1 protein from the African prototype S27 strain (UniProt Accession no. Q8JUX5) was synthesized (Epoch Biosciences) and cloned in the VSV antigenome plasmid to replace its native glycoprotein G as previously reported [37]. The VSV genome also carries an enhanced green fluorescent proteion (eGFP) marker to score infected cells. A plasmid-based rescue system was used to generate rVSV-CHIKV [52]. Rescued virus was grown on Vero cells and Sanger sequencing was used to confirm the glycoprotein gene sequence.

### Fusion and post-attachment assays with CHIKV 181/25

For the fusion assay, Vero cells were seeded in 96-well plates, washed with binding medium (RPMI 1640 prepared without sodium bicarbonate, 0.2% BSA, 10 mM HEPES pH 7.4, and 20 mM NH_4_Cl), and incubated at 4°C for 15 minutes. CHIKV181/25 prepared in binding medium was added to cells and allowed to bind for 1 hour at 4°C with shaking. Cells were washed with binding buffer and mAbs diluted in cold DMEM with 2% FBS were added and incubated at 4°C for 1 hour. Cells were then treated with fusion media (RPMI 1640 prepared without sodium bicarbonate, 0.2% BSA, 10 mM HEPES pH 5.5) and incubated at 37°C for 2 minutes. Fusion media was removed and replaced with DMEM with 2% FBS and 20mM NH_4_Cl. Cells were incubated for 6 hours at 37°C, overlaid with methylcellulose for 16 hours and the FRNT protocol was followed. Dependence of viral entry on low pH-mediated membrane fusion was confirmed by comparison to cells treated with control media (RPMI 1640, 0.2% BSA, 10 mM HEPES pH 7.4), which exhibited infectivity of less than 25% relative to low-pH treated cells.

For the post-attachment assay, Vero cells were seeded with 2.5 x 10^4^ cells/well overnight in 96-well plates. Cells were pre-chilled at 4°C for 15 minutes before 150 FFU of CHIKV181/25 was allowed to bind cells for 1 hour at 4°C. Cells were washed with DMEM with 2% FBS to remove free virus, and diluted mAbs were added and incubated for 1 hour at 4°C. Virus was allowed to internalize for 15 minutes at 37°C, cells were overlaid with methylcellulose for 16 hours, and the FRNT protocol was followed as above.

### rVSV-CHIKV neutralization assay and escape mutant generation

For antibody neutralization experiments, pre-titrated amounts of rVSV-CHIKV particles were incubated with increasing concentrations of test antibody at 37°C for 1 h prior to addition to cell monolayers in 96-well plates. After 1 h of infection, 20 mM of NH_4_Cl was added to halt subsequent rounds of infection. The infection rate of rVSV-CHIKV was measured by automated enumeration of eGFP+ cells (infectious units) using a Cell Insight CX5 imager (Thermo Fisher) at 16 h post-infection.

Escape mutant selections were performed by serial passage of rVSV-CHIKV particles in the presence of test mAb. Serial 10-fold dilutions of virus were preincubated with a concentration of mAb corresponding to the IC90 value derived from neutralization assays, and then added to 70% confluent monolayers of Vero cells in 12-well plates, in duplicate. Infection was allowed to proceed to completion (> 90% cell death by eye), and supernatants were harvested from the infected wells that received the highest dilution (*i*.*e*., the least amount) of viral inoculum.

Following three to four subsequent passages under mAb selection with virus-containing supernatants as above, supernatants were tested for viral neutralization escape. If viral populations demonstrated resistance to test antibody, individual viral clones were plaque-purified on Vero cells, and amplified for sequencing. Viral RNA isolation was performed on each viral clone using Viral RNA Kit (Zymo research) and cDNA synthesis was performed. Glycoprotein gene was amplified by using primers flanking the upstream and downstream of CHIKV glycoprotein and subsequently sequenced.

### *In vivo* challenge with CHIKV LR2006_OPY1

Subcutaneous injections in the footpad were performed under anesthesia that was induced and maintained with ketamine hydrochloride and xylazine, and all efforts were made to minimize suffering. MAbs (100 μg in PBS, 6 mg/kg) were administered to 3-week-old male C57BL/6 mice treated with 0.25 mg of an anti-Ifnar1 blocking mouse MAb (MAR1-5A3) [44] via intraperiontal injection 1 day prior to or following CHIKV-LR inoculation. Mice were inoculated subcutaneously with 10^3^ FFU of CHIKV-LR diluted in PBS and survival followed for 21 days.

### Pharmacokinetic studies in uninfected mice

Eight to ten week-old ICR mice (n = 3) received 100 μg/mouse of antibody intravenously on Day 0. Blood draws were obtained on days 3 and 6. Serum collected was then evaluated by ELISA to detect human IgG. Mouse serum samples were tested using a commercial ELISA (Abcam cat No. ab100547) for quantifying human IgG. Data was analyzed and graphed using GraphPad Prism v6.0.

### Negative stain electron microscopy

800 pmol of purified DC2.271B Fab was mixed with 100 pmol purified p62/E1 and incubated overnight at 4°C. The resulting complex was recovered by size-exclusion chromatography using an S200i column (GE Healthcare, IL) mounted on a fast protein liquid phase system (Äkta pure; GE Healthcare, IL). Pure antigen alone or purified Fab-antigen complex were deposited on plasma-cleaned (Gatan Solarus 950 Plasma system, CA) carbon-coated 400 mesh copper EM grids (Protochips Inc, NC) and embedded in 2% w/V uranyl formate. The resulting p62/E1 nsEM specimen was introduced into an FEI Tecnai G2 F20 electron microscope mounted with a Tietz TemCamCF416 CMOS camera. Data was collected under low-dose conditions at 200 kV, 60,000X nominal magnification and 1 um nominal underfocus. The resulting data pixel size was 1.79 Å. Similarly, the p62-E1/DC2.271B Fab nsEM specimen was introduced into an FEI Tecnai T12 electron microscope mounted with a Tietz TemCamCF416 CMOS camera. Data were collected under low-dose conditions at 120 kV, 60,000X nominal magnification and 1 um nominal underfocus. The resulting data pixel size was 2.54 Å.

Contrast transfer functions for each micrograph were modeled using GCTF [53]. Both data sets were Fourier cropped by a factor of 2. Identification of particles in the micrographs was performed with a difference-of-Gaussian approach [54]. Particle images were extracted and reference-free 2D class averaging was performed correcting the data for microscope contrast transfer functions by phase flipping (Relion 3.0) [55]. Particles contributing to meaningful class averages were selected for further processing. A simulated density map (PDB ID 3N40) [9] was low-pass filtered to 40Å and used as reference for iterative Euler angle recovery and 3D object reconstruction of the data (Relion 3.0). Nominal FSC 0.5 resolutions of the resulting density maps were 16 Å (p62/E1) and 16 Å (p62-E1/DC2.271B Fab).

### Accession numbers

The EM reconstruction data have been deposited in the Electron Microscopy Data Bank under codes EMD-20268 (Chikungunya virus p62-E1) and EMD-20269 (Chikungunya virus p62-E1 in complex with Fab from IgG DC2.271B).

## Supporting information

S1 Fig Representative FACS sort of patient-derived PBMCs. Cells were filtered for size and granularity (**A**), then (in this case) CD3^+^/CD8^+^/CD14^+^ cells eliminated (**B**). The CD27^+^ / CD20^hi^ / IgG^+^ / p62-E1^+^ B cells (**C** and **D**) were collected in individual wells. In some samples, both CD20^hi/lo^ populations were carried forward.
(PDF)

S2 Fig Single-Point ELISA of mAbs (300 nM) against Wells Coated with BSA. Experiments performed as in **Fig 1D**.
(PDF)

S3 Fig Immunoprecipitation of viral proteins from infected cells. BHK-21 cells were inoculated with CHIK 181/25 for 8 h, labeled with [^35^S]methionine/cysteine for 2 h, and lysed on ice. Approximately 1 μg of the indicated mAbs or 2 μL of a control SFV polyclonal antibody [42] was incubated with lysate for 1 h in the presence of 0.1% SDS. The immunoprecipitate was retrieved with Protein A agarose, and the samples were reduced and alkylated and analyzed by SDS-PAGE and fluorography. The lower migrating immunoprecipitated protein is E2, the middle is E1, and the upper is p62 (arrows) as demonstrated by control antibodies (chCHK-166^pMAZ^:E1, chCHK-265^pMAZ^:E2/p62, and SFV polyclonal antibody:E2/p62). Only some mAbs resulted in an IP; data for all other mAbs were negative.
(PDF)

S4 Fig Binding of E1-Specific mAbs. (**A**) Initial screening data of mAbs for binding to E1' by ELISA at a single mAb concentration (300 nM). Only those mAbs having appreciable activity against E1' are shown, all others were negative. Each point represents the mean of two replicates. (**B**) BLI analysis of interactions between E1-specific mAbs and p62-E1. A representative dataset from two independent experiments is shown. (**C**) ELISA analysis of binding of E1-specific mAbs to E1', p62-E1, or BSA. For p62-E1 ELISA, only DC1.7, DC2.284, DC2.315, and DC2.415 were analyzed. A representative dataset from two independent experiments performed in triplicate is shown. Each point represents mean ± SD.
(PDF)

S5 Fig Competition studies for E1-Specific mAbs. The ability of chCHK-166^pMAZ^ to engage p62-E1/human mAb complexes was tested in two-phase BLI experiments. SUDV-F4 was included as a negative control. A representative dataset from two independent experiments is shown.
(PDF)

S6 Fig Neutralization curves of human mAbs against CHIKV 181/25. For each mAb, a representative dataset performed in triplicate from two or more independent experiments is shown (IC_50_ values were consistent among experiments). Points represent the mean ± SD. Curves are color-coded according to epitope designation (blue, p62-E1 specific; red, E1-specific; gray, undefined). The IC_50_ value is provided, along with 95% confidence interval from curve fitting (these values are also shown graphically in **Fig 3B** of the main text).
(PDF)

S7 Fig Screening of 35 mAbs for neutralizing activity against MAYV. mAbs were tested at 30 nM and 300 nM; chCHK-265^pMAZ^ and SUDV-F4 were included as controls. Experiments performed in triplicate, each bar represents mean ± SD.
(PDF)

S8 Fig Neutralization of rVSV-CHIKV by CHIKV mAbs. (**A**) Neutralization curve of rVSV-CHIKV by chCHK-152^pMAZ^. A representative dataset from three independent experiments each performed in triplicate is shown. Points represent mean ± SD. (**B**) Images of infected cells at low and high concentrations of DC2.271B, DC2.429, DC1.7, and DC2.315. Quantification of infected cells at several concentrations led to the neutralization curves shown in **Figs 4B** and **5B**
(PDF)

S9 Fig Alignment of CHIKV E1 sequences Near DIII. The domain boundaries are indicated at the bottom. The two positions at which viral escape mutations were isolated for E1-specific human mAbs (A286 for DC2.315, green, and R289 for DC1.7, cyan) are indicated.
(PDF)

S10 Fig SEC-HPLC analysis of mAbs. Antibodies were characterized by SEC-HPLC using a ProSEC 300S 300x7.5 mm column (Agilent Technologies) on a LC 1260 Infinity Series HPLC (Agilent Technologies). Column was equilibrated with 50 mM phosphate buffer, 150 mM NaCl, Ph 7.0 at 1.00 mL/min. Chromatographs and peak area percent are reported using Agilent Openlabs software.
(PDF)
