# Supplementary material for: Human monoclonal antibodies against chikungunya virus target multiple distinct epitopes in the E1 and E2 glycoproteins
Source: PLoS Pathog. 2019 Nov 7;15(11):e1008061. doi: 10.1371/journal.ppat.1008061 (PMC6837291; doi:10.1371/journal.ppat.1008061)
Supplement: S1 Fig — Cells were filtered for size and granularity (A), then (in this case) CD3+/CD8+/CD14+ cells eliminated (B). The CD27+ / CD20hi / IgG+ / p62-E1+ B cells (C and D) were collected in individual wells. In some samples, both CD20hi/lo populations were carried forward. (PDF) [file ppat.1008061.s001.pdf]

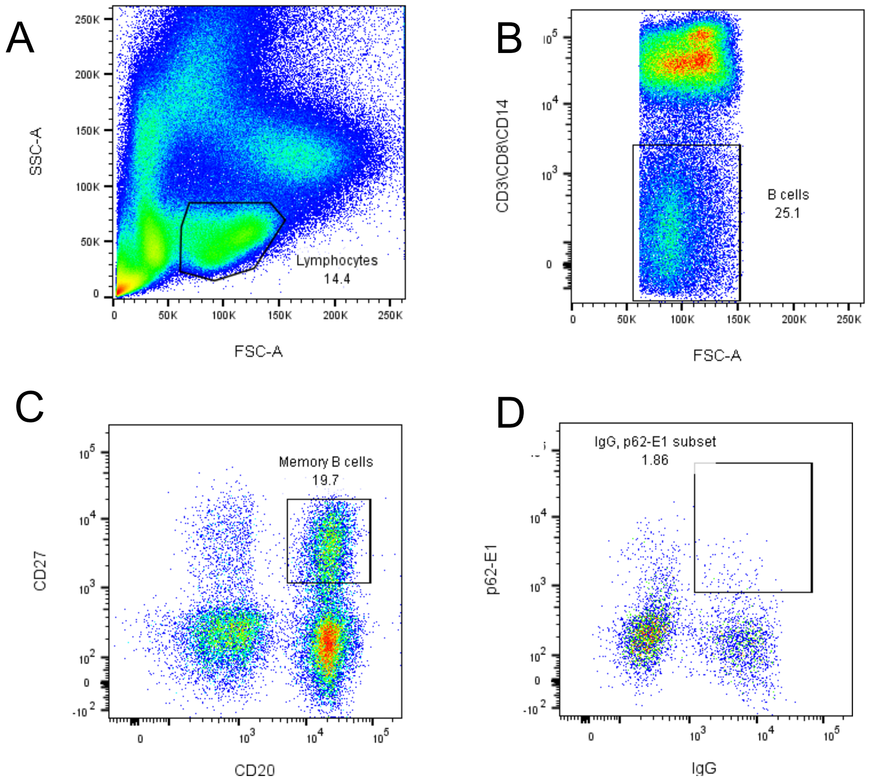

Figure S1. Representative FACS Sort of Patient-Derived PBMCs. Cells were filtered for size and granularity (**A**), then (in this case) $CD3^+/CD8^+/CD14^+$ cells eliminated (**B**). The $CD27^+$ / $CD20^{hi}$ / IgG^+ / $p62\text{-}E1^+$ B cells (**C** and **D**) were collected in individual wells. In some samples, both $CD20^{hi/lo}$ populations were carried forward.
