# Supplementary material for: Human monoclonal antibodies against chikungunya virus target multiple distinct epitopes in the E1 and E2 glycoproteins
Source: PLoS Pathog. 2019 Nov 7;15(11):e1008061. doi: 10.1371/journal.ppat.1008061 (PMC6837291; doi:10.1371/journal.ppat.1008061)
Supplement: S2 Fig — Experiments performed as in Fig 1D. (PDF) [file ppat.1008061.s002.pdf]

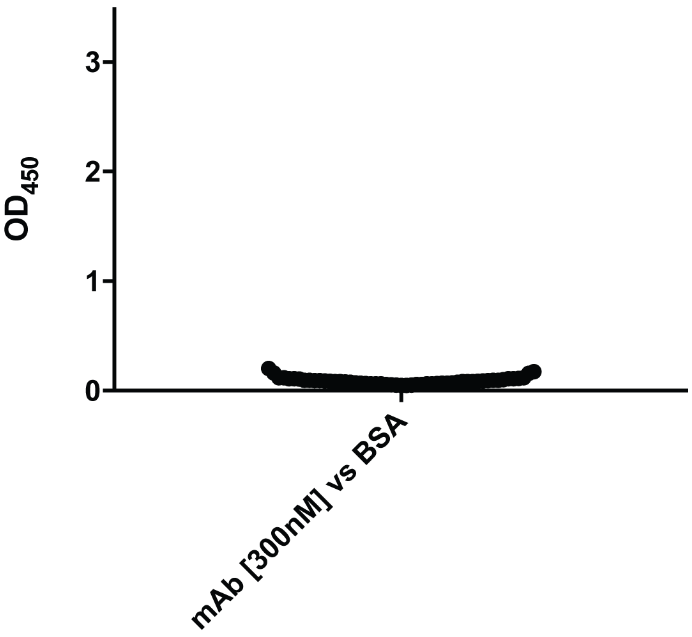

Figure S2. Single-Point ELISA of mAbs (300 nM) against Wells Coated with BSA. Experiments performed as in **Fig. 1D**.
