# Supplementary material for: Human monoclonal antibodies against chikungunya virus target multiple distinct epitopes in the E1 and E2 glycoproteins
Source: PLoS Pathog. 2019 Nov 7;15(11):e1008061. doi: 10.1371/journal.ppat.1008061 (PMC6837291; doi:10.1371/journal.ppat.1008061)
Supplement: S3 Fig — BHK-21 cells were inoculated with CHIK 181/25 for 8 h, labeled with [35S]methionine/cysteine for 2 h, and lysed on ice. Approximately 1 μg of the indicated mAbs or 2 μL of a control SFV polyclonal antibody [42] was incubated with lysate for 1 h in the presence of 0.1% SDS. The immunoprecipitate was retrieved with Protein A agarose, and the samples were reduced and alkylated and analyzed by SDS-PAGE and fluorography. The lower migrating immunoprecipitated protein is E2, the middle is E1, and the upper is p62 (arrows) as demonstrated by control antibodies (chCHK-166pMAZ:E1, chCHK-265pMAZ:E2/p62, and SFV polyclonal antibody:E2/p62). Only some mAbs resulted in an IP; data for all other mAbs were negative. (PDF) [file ppat.1008061.s003.pdf]

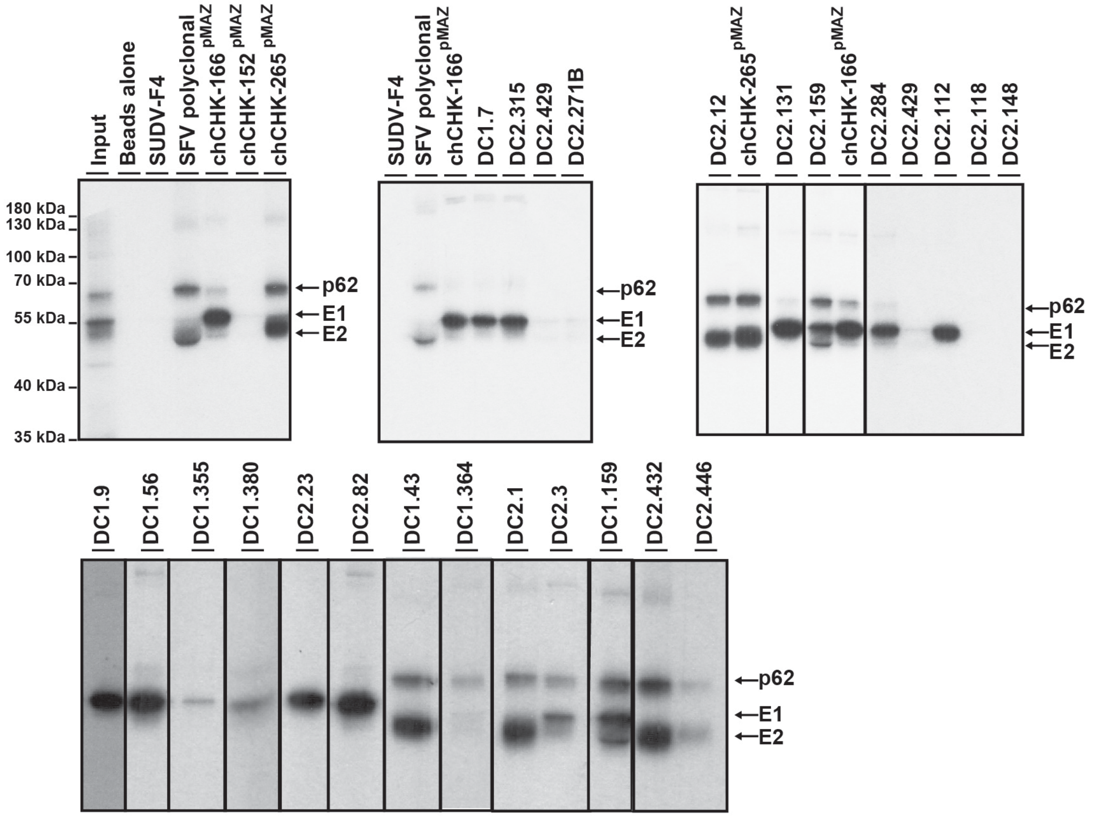

Figure S3. Immunoprecipitation of viral proteins from infected cells. BHK-21 cells were inoculated with CHIK 181/25 for 8 h, labeled with [^{35}S]methionine/cysteine for 2 h, and lysed on ice. Approximately 1 μg of the indicated mAbs or 2 μL of a control SFV polyclonal antibody (Sanchez-San Martin et al., 2013) was incubated with lysate for 1 h in the presence of 0.1% SDS. The immunoprecipitate was retrieved with Protein A agarose, and the samples were reduced and alkylated and analyzed by SDS-PAGE and fluorography. The lower migrating immunoprecipitated protein is E2, the middle is E1, and the upper is p62 (arrows) as demonstrated by control antibodies (chCHK-166^{pMAZ}:E1, chCHK-265^{pMAZ}:E2/p62, and SFV polyclonal antibody:E2/p62). Only some mAbs resulted in an IP; data for all other mAbs were negative. Reference: Sanchez-San Martin, C., Nanda, S., Zheng, Y., Fields, W., and Kielian, M. (2013). Cross-inhibition of chikungunya virus fusion and infection by alphavirus E1 domain III proteins. Journal of virology *87*, 7680-7687.
