# Supplementary material for: Human monoclonal antibodies against chikungunya virus target multiple distinct epitopes in the E1 and E2 glycoproteins
Source: PLoS Pathog. 2019 Nov 7;15(11):e1008061. doi: 10.1371/journal.ppat.1008061 (PMC6837291; doi:10.1371/journal.ppat.1008061)
Supplement: S4 Fig — (A) Initial screening data of mAbs for binding to E1' by ELISA at a single mAb concentration (300 nM). Only those mAbs having appreciable activity against E1' are shown, all others were negative. Each point represents the mean of two replicates. (B) BLI analysis of interactions between E1-specific mAbs and p62-E1. A representative dataset from two independent experiments is shown. (C) ELISA analysis of binding of E1-specific mAbs to E1', p62-E1, or BSA. For p62-E1 ELISA, only DC1.7, DC2.284, DC2.315, and DC2.415 were analyzed. A representative dataset from two independent experiments performed in triplicate is shown. Each point represents mean ± SD. (PDF) [file ppat.1008061.s004.pdf]

A

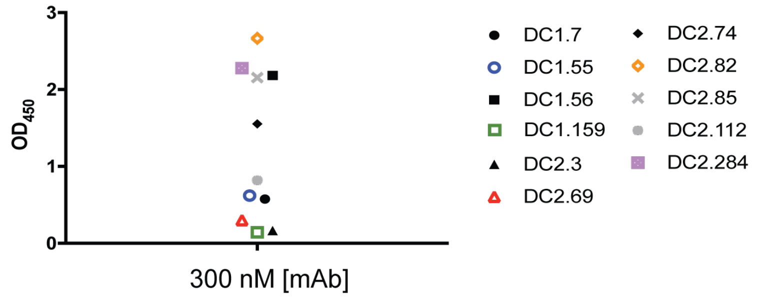

B

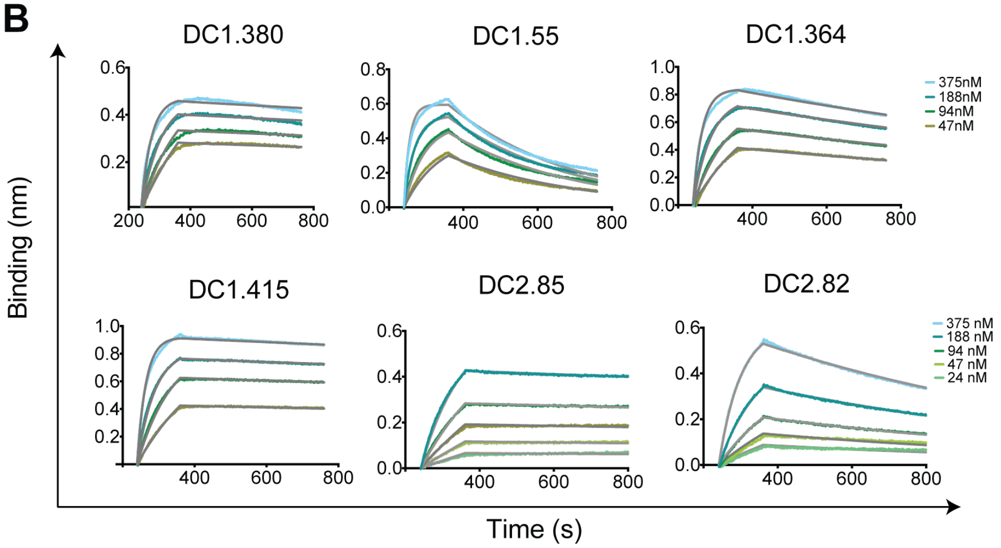

mAb	k_{on} ($M^{-1}s^{-1}$)	k_{off} (s^{-1})	K_D (M)
DC 1.380	$(8.16\pm0.07)\times10^{4}$	$(1.67\pm0.05)\times10^{-4}$	$(2.04\pm0.07)\times10^{-9}$
DC 1.55	$(1.72\pm0.02)\times10^{5}$	$(2.99\pm0.01)\times10^{-3}$	$(1.74\pm0.02)\times10^{-8}$
DC 1.364	$(1.08\pm0.07)\times10^{5}$	$(6.04\pm0.04)\times10^{-4}$	$(5.57\pm0.05)\times10^{-9}$
DC 1.415	$(1.10\pm0.05)\times10^{5}$	$(1.28\pm0.03)\times10^{-4}$	$(1.17\pm0.03)\times10^{-9}$
DC 2.85	$(4.68\pm0.02)\times10^{4}$	$(1.44\pm0.02)\times10^{-4}$	$(3.07\pm0.04)\times10^{-9}$
DC 2.82	$(4.34\pm0.03)\times10^{4}$	$(1.04\pm0.03)\times10^{-3}$	$(2.39\pm0.02)\times10^{-8}$

C

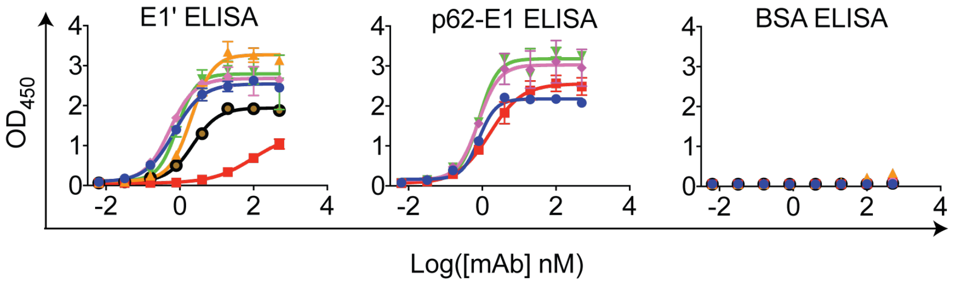

	EC_{50} (nM) (95% C.I.)	
	E1’	p62-E1
DC1.7	0.69 (0.57-0.83)	0.80 (0.65-0.94)
DC1.56	2.0 (1.7-2.4)	ND
DC2.284	0.53 (0.40-0.68)	0.78 (0.50-1.1)
DC2.315	>	1.6 (1.1-2.2)
DC1.415	0.86 (0.58-1.2)	0.81 (0.56-1.1)
chCHK-166^{pMAZ}	2.2 (1.9-2.6)	ND

Figure S4. Binding of E1-Specific mAbs. (**A**) Initial screening data of mAbs for binding to E1' by ELISA at a single mAb concentration (300 nM). Only those mAbs having appreciable activity against E1' are shown, all others were negative. Each point represents the mean of two replicates. (**B**) BLI analysis of interactions between E1-specific mAbs and p62-E1. A representative dataset from two independent experiments is shown. (**C**) ELISA analysis of binding of E1-specific mAbs to E1', p62-E1, or BSA. For p62-E1 ELISA, only DC1.7, DC2.284, DC2.315, and DC2.415 were analyzed. A representative dataset from two independent experiments performed in triplicate is shown. Each point represents mean ± SD.
