# Supplementary material for: Human monoclonal antibodies against chikungunya virus target multiple distinct epitopes in the E1 and E2 glycoproteins
Source: PLoS Pathog. 2019 Nov 7;15(11):e1008061. doi: 10.1371/journal.ppat.1008061 (PMC6837291; doi:10.1371/journal.ppat.1008061)
Supplement: S5 Fig — The ability of chCHK-166pMAZ to engage p62-E1/human mAb complexes was tested in two-phase BLI experiments. SUDV-F4 was included as a negative control. A representative dataset from two independent experiments is shown. (PDF) [file ppat.1008061.s005.pdf]

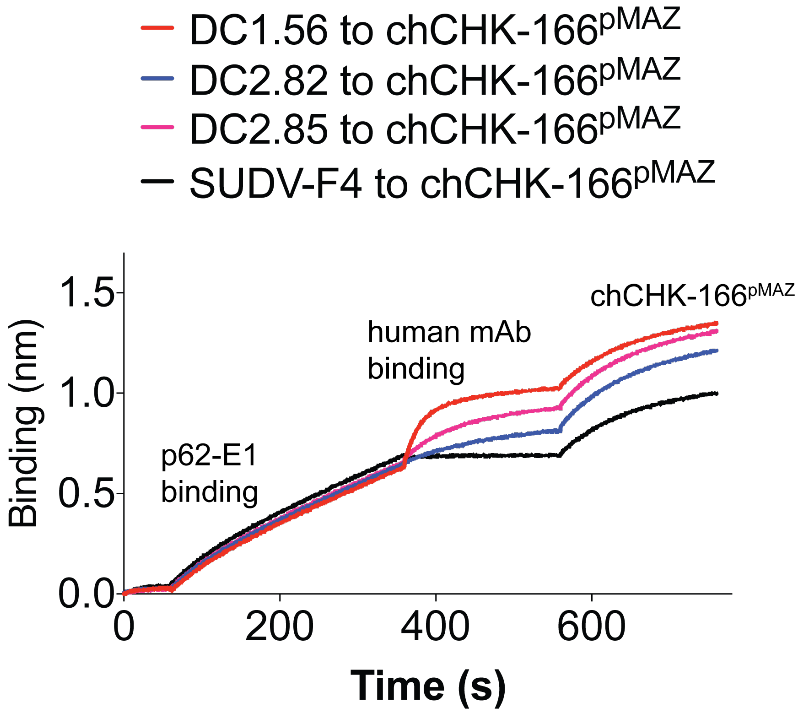

Figure S5. Competition Studies for E1-Specific mAbs. The ability of chCHK-166^{pMAZ} to engage p62-E1/human mAb complexes was tested in two-phase BLI experiments. SUDV-F4 was included as a negative control. A representative dataset from two independent experiments is shown.
