# Supplementary material for: Human monoclonal antibodies against chikungunya virus target multiple distinct epitopes in the E1 and E2 glycoproteins
Source: PLoS Pathog. 2019 Nov 7;15(11):e1008061. doi: 10.1371/journal.ppat.1008061 (PMC6837291; doi:10.1371/journal.ppat.1008061)
Supplement: S6 Fig — For each mAb, a representative dataset performed in triplicate from two or more independent experiments is shown (IC50 values were consistent among experiments). Points represent the mean ± SD. Curves are color-coded according to epitope designation (blue, p62-E1 specific; red, E1-specific; gray, undefined). The IC50 value is provided, along with 95% confidence interval from curve fitting (these values are also shown graphically in Fig 3B of the main text). (PDF) [file ppat.1008061.s006.pdf]

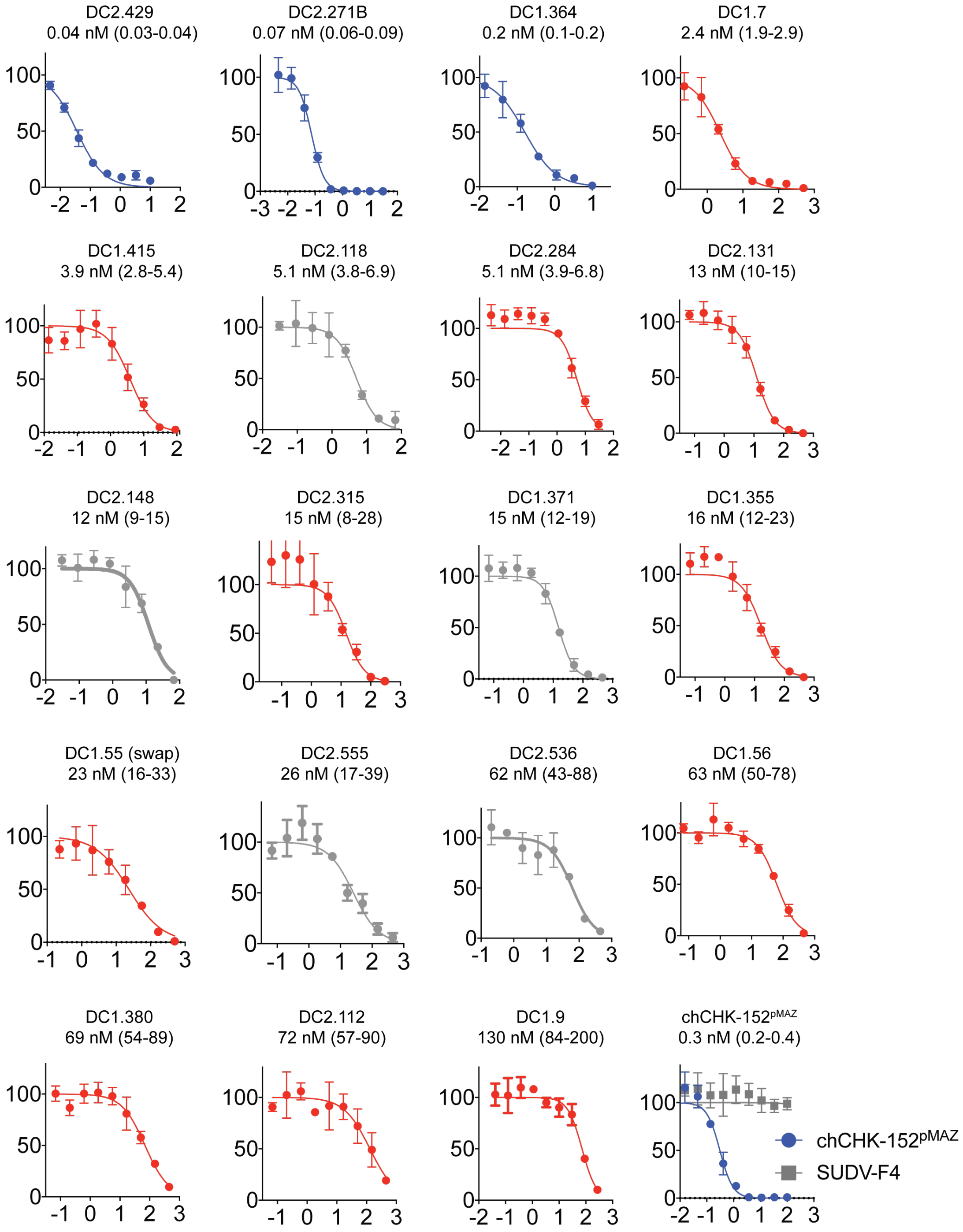

Figure S6. Neutralization Curves of Human mAbs against CHIKV 181/25. For each mAb, a representative dataset performed in triplicate from two or more independent experiments is shown (IC_{50} values were consistent among experiments). Points represent the mean ± SD.

Curves are color-coded according to epitope designation (blue, p62-E1 specific; red, E1-specific; gray, undefined). The IC_{50} value is provided, along with 95% confidence interval from curve fitting (these values are also shown graphically in **Fig. 3B** of the main text).
