# Supplementary material for: Human monoclonal antibodies against chikungunya virus target multiple distinct epitopes in the E1 and E2 glycoproteins
Source: PLoS Pathog. 2019 Nov 7;15(11):e1008061. doi: 10.1371/journal.ppat.1008061 (PMC6837291; doi:10.1371/journal.ppat.1008061)
Supplement: S7 Fig — mAbs were tested at 30 nM and 300 nM; chCHK-265pMAZ and SUDV-F4 were included as controls. Experiments performed in triplicate, each bar represents mean ± SD. (PDF) [file ppat.1008061.s007.pdf]

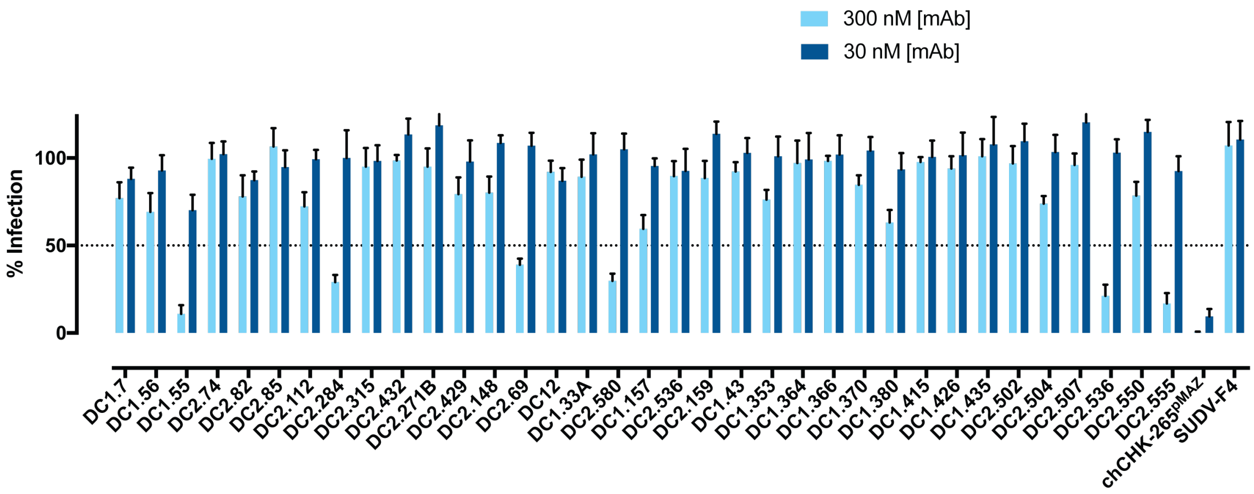

Figure S7. Screening of 35 mAbs for Neutralizing Activity against MAYV. mAbs were tested at 30 nM and 300 nM; chCHK-265^{pMAZ} and SUDV-F4 were included as controls. Experiments performed in triplicate, each bar represents mean ± SD.
