# Supplementary material for: Human monoclonal antibodies against chikungunya virus target multiple distinct epitopes in the E1 and E2 glycoproteins
Source: PLoS Pathog. 2019 Nov 7;15(11):e1008061. doi: 10.1371/journal.ppat.1008061 (PMC6837291; doi:10.1371/journal.ppat.1008061)
Supplement: S8 Fig — (A) Neutralization curve of rVSV-CHIKV by chCHK-152pMAZ. A representative dataset from three independent experiments each performed in triplicate is shown. Points represent mean ± SD. (B) Images of infected cells at low and high concentrations of DC2.271B, DC2.429, DC1.7, and DC2.315. Quantification of infected cells at several concentrations led to the neutralization curves shown in Figs 4B and 5B (PDF) [file ppat.1008061.s008.pdf]

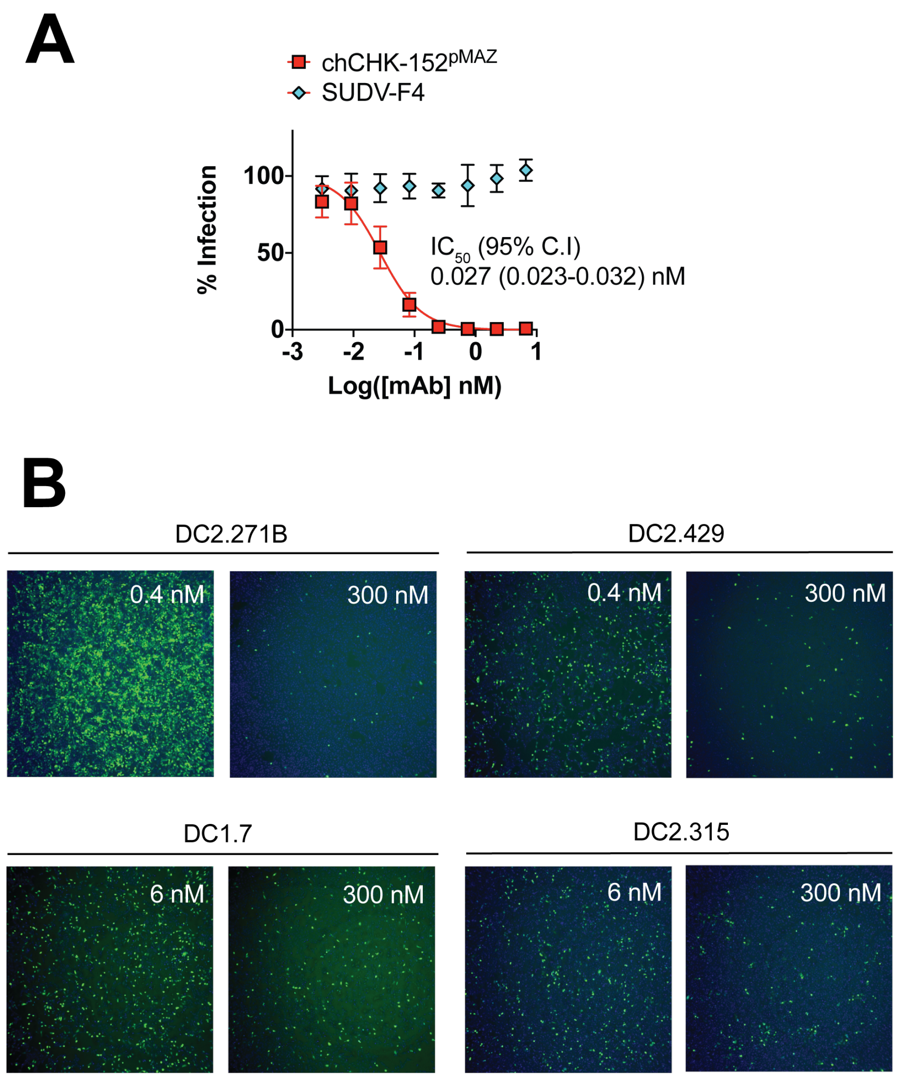

Figure S8. Neutralization of rVSV-CHIKV by CHIKV mAbs. (**A**) Neutralization curve of rVSV-CHIKV by chCHK-152^{pMAZ}. A representative dataset from three independent experiments each performed in triplicate is shown. Points represent mean ± SD. (**B**) Images of infected cells at low and high concentrations of DC2.271B, DC2.429, DC1.7, and DC2.315. Quantification of infected cells at several concentrations led to the neutralization curves shown in **Figs. 4B** and **5B**
