# Supplementary material for: Human monoclonal antibodies against chikungunya virus target multiple distinct epitopes in the E1 and E2 glycoproteins
Source: PLoS Pathog. 2019 Nov 7;15(11):e1008061. doi: 10.1371/journal.ppat.1008061 (PMC6837291; doi:10.1371/journal.ppat.1008061)
Supplement: S9 Fig — The domain boundaries are indicated at the bottom. The two positions at which viral escape mutations were isolated for E1-specific human mAbs (A286 for DC2.315, green, and R289 for DC1.7, cyan) are indicated. (PDF) [file ppat.1008061.s009.pdf]

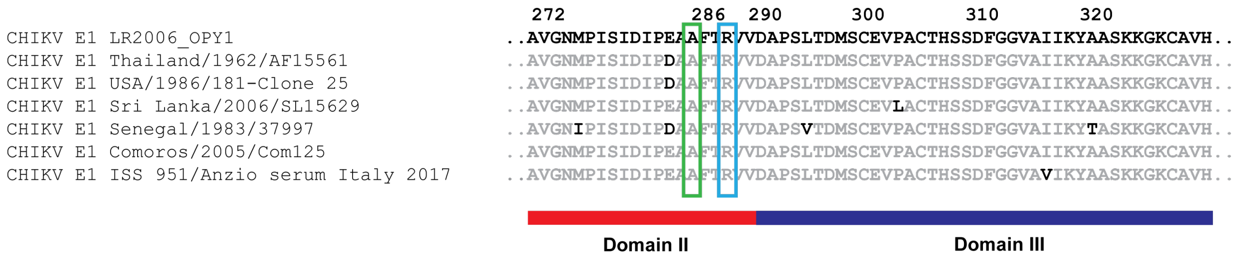

Figure S9. Alignment of CHIKV E1 Sequences Near DIII. The domain boundaries are indicated at the bottom. The two positions at which viral escape mutations were isolated for E1-specific human mAbs (A286 for DC2.315, green, and R289 for DC1.7, cyan) are indicated.
