# Supplementary material for: Human monoclonal antibodies against chikungunya virus target multiple distinct epitopes in the E1 and E2 glycoproteins
Source: PLoS Pathog. 2019 Nov 7;15(11):e1008061. doi: 10.1371/journal.ppat.1008061 (PMC6837291; doi:10.1371/journal.ppat.1008061)
Supplement: S10 Fig — Antibodies were characterized by SEC-HPLC using a ProSEC 300S 300x7.5 mm column (Agilent Technologies) on a LC 1260 Infinity Series HPLC (Agilent Technologies). Column was equilibrated with 50 mM phosphate buffer, 150 mM NaCl, Ph 7.0 at 1.00 mL/min. Chromatographs and peak area percent are reported using Agilent Openlabs software. (PDF) [file ppat.1008061.s010.pdf]

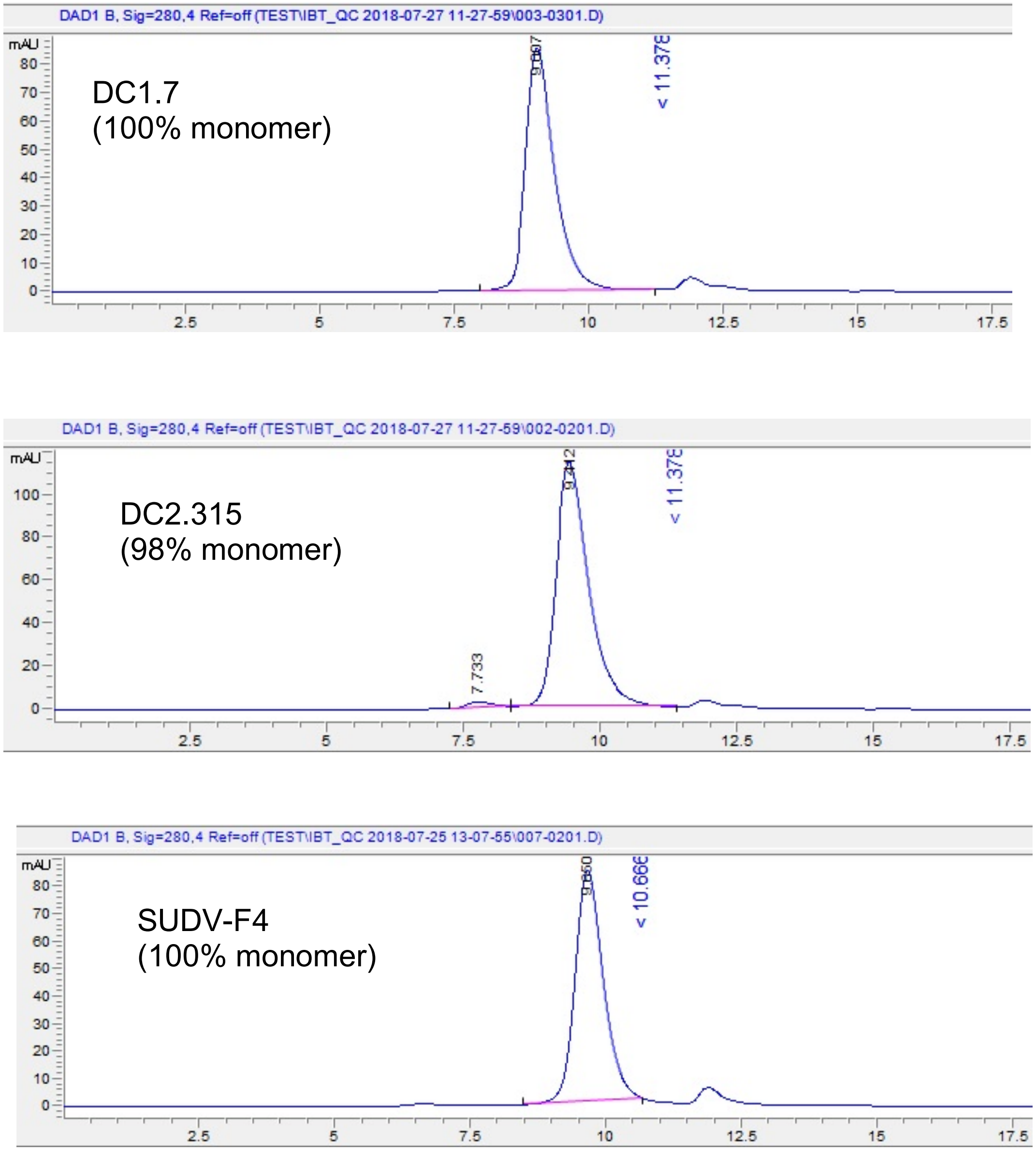

Figure S10. SEC-HPLC Analysis of mAbs. Antibodies were characterized by SEC-HPLC using a ProSEC 300S 300x7.5 mm column (Agilent Technologies) on a LC 1260 Infinity Series HPLC (Agilent Technologies). Column was equilibrated with 50 mM phosphate buffer, 150 mM NaCl, Ph 7.0 at 1.00 mL/min. Chromatographs and peak area percent are reported using Agilent Openlabs software.
